# Biomedical applications of organoids derived from the digestive system

**DOI:** 10.3389/fcell.2025.1599384

**Published:** 2025-05-30

**Authors:** Zhensheng Xu, Zhongwen Lei, Qiuhua Cheng, Yuanhui Gao, Yang Xiang

**Affiliations:** ^1^ Department of Oncologic Chemotheraphy, Haikou Affiliated Hospital of Central South University Xiangya School of Medcine, Haikou, China; ^2^ Department of Hepatobiliary Surgery, Haikou Affiliated Hospital of Central South University Xiangya School of Medcine, Haikou, China; ^3^ Central Laboratory, Haikou Affiliated Hospital of Central South University Xiangya School of Medcine, Haikou, China

**Keywords:** digestive system, organoids, mechanisms of growth, modeling of disease, drug screening, regenerative medicine

## Abstract

The global incidence of digestive system diseases is increasing, posing a significant public health challenge and driving an escalating demand for research into the mechanisms underlying their onset and progression. Traditional cell models and xenotransplantation animal models have been widely used to simulate human digestive diseases, thereby enhancing our understanding of disease occurrence, progression, and drug resistance. However, these models fail to fully replicate the complex cellular microenvironment and spatial structure, and are further limited by individual and species differences. Organoid technology, as an emerging *in vitro* cell culture approach, enables the precise culturing and differentiation of human stem cells to generate highly tissue-specific and functionally intact organoids. This technology not only better recapitulates cell-to-cell interactions, extracellular matrix (ECM) microenvironment, and organ-specific physiological functions but also more closely mimics the human physiological state *in vitro*. Moreover, it reduces reliance on animal experiments, enhances the translatability of research findings, mitigates the limitations of animal models and two-dimensional cell models, and plays a pivotal role in simulating the physiological and pathological processes of the human digestive tract. Currently, common techniques for constructing organoids include embedding culture, rotating culture, magnetic suspension culture, organ-on-a-chip, three-dimensional (3D), and four-dimensional (4D) printing technologies. Seed cells are primarily derived from digestive system epithelial cells and pluripotent stem cells. This article reviews the construction methods of digestive system organoids, evaluates their applications in studying growth and development mechanisms, disease modeling and mechanism research, drug screening, regenerative medicine, and precision medicine, and identifies existing challenges and future research directions to provide a valuable reference for biomedical research.

## 1 Introduction

The digestive system primarily consists of the digestive tract and associated digestive glands. The digestive tract encompasses the oral cavity, pharynx, esophagus, stomach, small intestine, and large intestine, while the principal digestive glands include the salivary glands, liver, and pancreas. These components play crucial roles in nutrient absorption, metabolism, and excretion within the human body. Research models for studying the digestive system typically involve animal models and two-dimensional (2D) cell cultures. These models facilitate our understanding of cellular signaling pathways in digestive diseases, guide drug design principles, identify potential therapeutic targets, and elucidate disease pathogenesis, thereby serving as indispensable tools in global biomedical research. However, animal models exhibit interspecies differences and individual variability, which may limit their translational relevance to humans. Meanwhile, 2D cell cultures fail to replicate the complex *in vivo* microenvironment and cannot adequately simulate three-dimensional cellular interactions, potentially leading to discrepancies in biological processes that do not accurately reflect *in vivo* conditions. This discrepancy can compromise the precision of experimental outcomes (Kim et al., 2020). Consequently, addressing the challenges posed by species, cellular, and organ-level differences in current biological research models is imperative.

Due to their origin from stem cells and their highly realistic three-dimensional structure and function, organoid models effectively reduce the limitations found in animal models and two-dimensional cell cultures. As a result, they hold significant application potential in digestive system studies. Organoids serve as tissue-like structures with specific spatial arrangements, created by culturing stem or progenitor cells in a three-dimensional environment *in vitro*. The key features of these models lie in the ability of stem/progenitor cells to undergo self-differentiation and self-organization. They can be utilized in various bioreactors, such as stirred tank reactors, microfluidic bioreactors, and perfusion-based systems, which facilitate the simulation of *in vitro* organ growth and development within a controlled microenvironment, ultimately leading to the differentiation into functional tissues/organs (Licata et al., 2023; O'Connell and Winter 2020).

**FIGURE 1:**
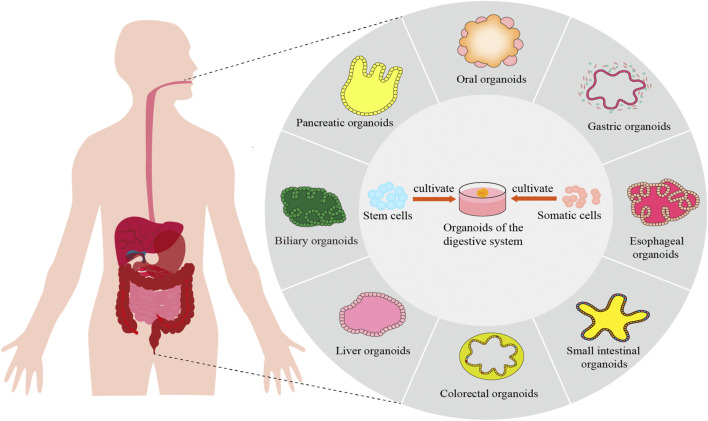
Construction and application of digestive system organoids.

Currently, the seed cells utilized in organoid cultures primarily consist of somatic cells and stem cells, with particular emphasis on pluripotent stem cells (PSCs) and adult stem cells (ASCs) (Tang et al., 2022). ASCs are advantageous due to their diverse sources, including diseased tissues, which can be cultured into patient-derived organoids (PDOs). PDOs exhibit genetic characteristics closely resembling those of patient tissues, making them highly promising for drug screening and personalized treatment in the digestive system (Yu et al., 2022). PSCs can be further categorized into embryonic stem cells (ESCs) and induced pluripotent stem cells (iPSCs). Organoids derived from PSCs replicate the early stages of organ development, with structural differentiation that closely mirrors fetal tissue (Nikokiraki et al., 2022). This review highlights recent advancements in gastrointestinal organoids, focusing on their engineering and biomedical applications (Figure 1). Organoid construction technologies encompass traditional embedding methods, rotating culture techniques, hanging drop cultures, as well as emerging technologies such as organ-on-a-chip systems, three-dimensional (3D) and four-dimensional (4D) printing. This paper reviews the research progress of organoids derived from ASCs or PSCs in various digestive organs, including the oral cavity, esophagus, stomach, small intestine, colorectum, digestive glands, liver, and pancreas. Additionally, it discusses the construction technologies of these organoids and their applications in disease modeling, mechanism studies, drug screening, and regenerative medicine, providing valuable insights for future research in the digestive system.

## 2 Construction of organoids in the digestive system

### 2.1 Common seed cells

The seed cell sources for digestive system organoids can be categorized into somatic cells and stem cells. Somatic cells primarily consist of epithelial cells from various parts of the digestive system, such as the intestine and liver. Among stem cells, ASCs and PSCs are extensively utilized.

Somatic cells possess a degree of stemness, allowing them to maintain their original tissue characteristics *in vitro* over extended periods with good genetic stability, making them suitable as seed cells for digestive system organoids (Fujii and Sato, 2021). In 2023, Hermans et al. successfully established stable tooth organoids using molar and incisor teeth from mice (Hermans et al., 2023). These organoids expressed dental epithelial stem cell markers and demonstrated the ability to differentiate into ameloblasts *in vitro*, providing a novel platform for studying tooth biology and development. Cancer cells can also serve as seed cells for establishing digestive system tumor organoids. Kasagi et al. successfully cultured esophageal organoids using the human esophageal cell line EPC2-hTERT, which replicated the natural differentiation process of esophageal epithelium (Kasagi et al., 2018). This study further revealed that Notch signaling promotes esophageal epithelial differentiation, while inhibiting this pathway impairs epithelial differentiation. Xu et al. obtained colorectal cancer tissue samples through surgical resection or endoscopic biopsy, washed them thoroughly, enzymatically digested them to form single tumor cells, embedded them in matrix gel, and cultured them for 7–10 days to generate colorectal organoids (Xu et al., 2018).

Adult stem cells are non-specialized cells located in developed tissues, exhibiting stem cell capabilities and existing within different tissues and organs throughout the body. As an example, Lgr5^+^ stem cells identified in the small intestine and colon can be employed to create organoids that replicate the structural and functional characteristics of natural tissue (Parente et al., 2024). By leveraging the intrinsic self-organization properties of intestinal epithelial stem cells (ISCs) and employing air-liquid interface culture in a minimally defined medium, Kwon et al. successfully induced ISCs to differentiate into intestinal epithelial organoids characterized by cellular diversity, villous structures, and barrier integrity, thereby providing a valuable tool for regenerative medicine and disease modeling (Kwon et al., 2024). Schumacher et al. developed gastric organoids containing diverse gastric epithelial cells, including chief and parietal cells, through co-culture of immortalized gastric mesenchymal cells with gastric epithelial stem cells, facilitating studies on damage repair and other functions of gastric epithelial cells (Schumacher et al., 2015). Basak et al. demonstrated that silencing Lgr5^+^ stem cells *in vitro* could be achieved through the inhibition of either the epidermal growth factor receptor (EGFR) or the mitogen-activated protein kinase (MAPK) signaling pathways, which subsequently promoted organoid development favoring enteroendocrine cell differentiation (Basak et al., 2017). Additionally, they found that concurrent suppression of Wnt, Notch, and MAPK signaling pathways facilitated the transformation of these organoids into various types of intestinal secretory cells (Basak et al., 2017). In a follow-up investigation, Fujii et al. refined the culture conditions for small intestinal organoids, showing that insulin-like growth factor 1 and fibroblast growth factor (FGF) 2 considerably boosted the clonogenic potential of human small intestinal stem cells, supporting both the self-renewal and multi-lineage differentiation capabilities of intestinal organoids (Fujii et al., 2018).

Pluripotent stem cells, such as human induced pluripotent stem cells (hiPSCs) or embryonic stem cells (hESCs), can be directed to differentiate into specific gastrointestinal epithelial cell types and ultimately form gastrointestinal organoids. hiPSCs have been utilized to generate a variety of gastrointestinal organoids, including those of the stomach, small intestine, and colon (Wang et al., 2022). Zhang et al. combined PSCs with retinoic acid and fibroblast growth factor (FGF) 10 in co-culture to establish salivary gland organoids. This method provides a robust model for studying salivary gland development *in vitro* and developing novel cell therapies (Zhang et al., 2022). Zhang et al. exposed RUES2-derived embryonic stem cells to a range of growth factors, such as activin A, FGF2, BMP4, and the Rho kinase inhibitor Y-27632. This exposure facilitated their differentiation into anterior foregut progenitors by suppressing the BMP, transforming growth factor (TGF)-β1, and Wnt signaling pathways. The resulting esophageal progenitor cells were subsequently cultivated into esophageal organoids (Zhang et al., 2018). Furthermore, directed differentiation can also be achieved through cell co-culture systems. For instance, co-culturing hepatocytes with mesenchymal stem cells or stellate cells can produce liver-like tissues (Afonso et al., 2024; Christine Verawaty Sibuea, 2020), while combining mesenchymal stem cells, iPSCs, and endothelial cells in co-culture can create vascularized liver organoids, which is advantageous for constructing larger organoids (Abbasalizadeh et al., 2023). In addition, to further replicate the complexity of the intestinal microenvironment, researchers have established a range of gastrointestinal organoid co-culture systems, including those involving immune cells, mesenchymal cells, or gut microbiota (Al-Qadami et al., 2025; Flood et al., 2024). These co-culture systems enable the simulation of intricate cell-cell and host-microbe interactions within the gut, thereby offering novel insights into the investigation of inflammatory bowel diseases and infectious diseases.

### 2.2 Several common build techniques

**FIGURE 2:**
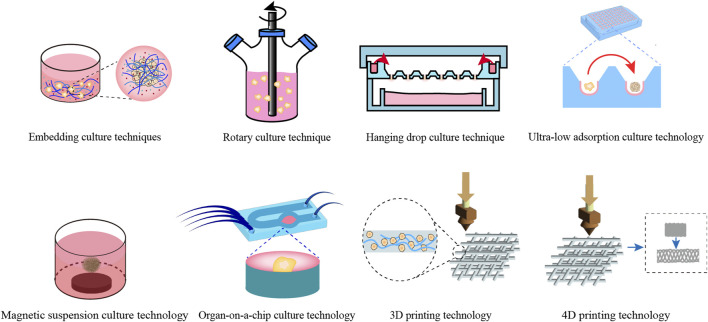
Construction techniques for common digestive system organoids. This figure offers a comprehensive overview of the conventional techniques utilized in the construction of digestive system organoids.

The construction technology for digestive system organoids can be categorized into traditional and novel methodologies. Traditional methodologies typically encompass embedding culture, rotary culture, hanging drop culture, magnetic levitation culture, and ultra-low attachment culture techniques, with embedding culture being the most prevalent. Novel construction technologies primarily consist of organ-on-a-chip, 3D printing, and 4D printing techniques (as illustrated in Figure 2).

#### 2.2.1 Traditional construction techniques

Traditional methods for constructing digestive system organoids primarily encompass the following areas.

##### 2.2.1.1 Embedding culture techniques

Embedding culture technology entails encapsulating cells within a matrix adhesive, subsequently incorporating various signaling proteins and growth factors to create an active three-dimensional framework (Habanjar et al., 2021). This method is distinguished by its ease of operation and gentle culturing environment. Nevertheless, the absence of direct cell-to-cell communication might impede the development of cell spheroids, and the expensive nature of the matrix adhesive presents obstacles for large-scale manufacturing (Lee et al., 2021).

Karakasheva et al. hydrolyzed esophageal tissue samples obtained via diagnostic biopsy or minimally invasive surgery using dispersing enzyme and trypsin, subsequently embedding the isolated cells in Matrigel matrix gel to form a single-cell suspension for 3D culture (Karakasheva et al., 2020). This method establishes a standardized protocol for esophageal organoid culture, serving as a valuable reference for other researchers. Matano et al. utilized recombinant human R-spondin1 (a Wnt pathway activator), epidermal growth factor, bone morphogenetic protein inhibitor Noggin, TGF-β1 receptor inhibitor A83-01, P38 inhibitor SB202190, and other growth factors in advanced DMEM/F12 medium to develop a colorectal organoid that can be cultured *in vitro* for extended periods (Matano et al., 2015).

##### 2.2.1.2 Rotation culture technique

Overall, the rotating cell culture system is utilized to maintain constant rotation of the cell culture medium, creating a microgravity environment that supports three-dimensional tissue formation (Mattei et al., 2019). This method improves the efficiency of nutrient uptake by cells and tissues, facilitating their growth and development. Nevertheless, it requires careful regulation of the rotation speed, as overly high speeds can harm cells and tissues, whereas insufficient speeds may cause sedimentation, hindering proper growth and development (Ryu et al., 2019).

He et al. effectively facilitated the self-differentiation and assembly of progenitor cells into hepatic bud-like organoids by culturing hollow hepatocyte-like organs in a rotary bioreactor under a dynamic suspension condition, thereby enhancing nutrient uptake and metabolic activity (He et al., 2022). Ye et al. developed a miniaturized rotary bioreactor called RPMotion and established tissue-specific settings and standard operating protocols for expanding human epithelial organoids derived from the liver, intestine, and pancreas. They observed that all organoid types proliferated significantly faster (5.2-fold, 3-fold, and 4-fold, respectively) in bioreactors compared to static cultures, while maintaining their organ-specific phenotypes. This advancement holds considerable promise for basic and translational research in gastrointestinal organoids (Ye et al., 2024).

As rotary culture technology advances in biliary tract applications, selecting appropriate rotary culture conditions becomes crucial for constructing digestive tract-like organs. This includes optimizing rotation speed, medium composition, and the addition of specific growth factors.

##### 2.2.1.3 Hanging drop culture technique

The hanging drop culture technique utilizes the surface tension and gravitational effects of inverted cell suspension droplets to form cell or tissue aggregates into spheroids at the liquid-air interface (Sun et al., 2021). This approach facilitates the efficient production of numerous uniform three-dimensional cellular spheroids, thus rendering it appropriate for industrial use. Nevertheless, because of the restricted volume of the droplets, the resulting spheroids are often relatively small in size (Zhou et al., 2023).

Price et al. developed an organoid hanging drop culture protocol that facilitates large-scale expansion and long-term maintenance of organoids using 5% Matrigel. They confirmed the genomic stability and phenotypic characteristics of these organoids, including drug sensitivity testing and clustered regularly interspaced short palindromic repeats (CRISPR-Cas9) genome-wide screening, with results consistent with those obtained under standard organoid culture conditions (Price et al., 2022). Hirokawa and colleagues developed a hanging drop culture system using a low-viscosity matrix (comprising 5% matrix glue). This system effectively supported the growth of organoids derived from both normal and tumor tissues obtained from colorectal cancer patients. Their research highlighted the effectiveness of this suspension-based approach for creating, maintaining, and developing organoid collections. Additionally, it showed promise for high-throughput drug screening and diagnostic evaluations involving tumor organoids (Hirokawa et al., 2021).

##### 2.2.1.4 Magnetic suspension culture technology

Magnetic levitation three-dimensional culture system is a technique wherein magnetized stem cells autonomously generate extracellular matrix to form organoids. Compared with traditional spheroid systems, the resulting organoids exhibit natural tissue-like characteristics and neuronal-dominated secretory functions, allowing for the rapid construction of functional organoids within a short timeframe (Marques et al., 2022). This approach allows for the manipulation of cell aggregate geometry using magnetic fields and supports the co-culture of various cell types. Nevertheless, it cannot replace the cell medium and encounters difficulties in regulating the size of cell aggregates, restricting its real-world applications (Tepe et al., 2023).

Adapikar et al. utilized suspension culture technology to cultivate taste stem/progenitor cells from the posterior tongue of mice, producing taste bud organoids. Compared with Matrigel-embedded organoids, these organoids possess functional taste receptor cells and circulating progenitor cells, demonstrating comparable differentiation and renewal rates to *in vivo* taste buds. Additionally, they maintain the capacity for taste receptor function and innervation by taste nerves, making them an excellent model for taste bud research (Adpaikar et al., 2022).

##### 2.2.1.5 Ultra-low adsorption culture technology

This approach utilizes ultra-low adsorption materials to prevent seed cells from adhering, promoting their assembly into spheroidal structures. Generally, 96-well and 384-well plates are well-suited for high-throughput three-dimensional cell cultures (Xing et al., 2024). The method is simple to execute and capable of generating cell spheroids with consistent diameters in large quantities. Additionally, the size of the spheroids can be regulated by modifying the number of initial seed cells. Nevertheless, this technique still demonstrates a relatively high variation coefficient (Ryu et al., 2019).

Kim et al. employed an ultra-low attachment culture method to develop hepatobiliary organoids that integrate both vascular and biliary components. The vascular network, which forms perfusable microvessels with lumens, enables these organoids to replicate liver diseases driven by interactions between parenchymal and nonparenchymal cells, showcasing potential applications (Kim et al., 2023). Chi et al. established multilineage liver organoids through the long-term expansion of cystic liver organoids derived from human pluripotent stem cells using ultra-low adsorption culture techniques. These organoids display structural intricacy and functional maturity, such as the development of vascular networks within parenchymal lobular structures, bile secretion polarity, and the capacity to respond to fibrotic signals, making them a valuable *in vitro* disease modeling tool (Chi et al., 2025).

#### 2.2.2 New construction techniques

Traditional culture methods face specific challenges in the development of digestive system organoids, such as a prolonged operation period, higher expenses, and limited ability to control structural formation. These issues impede the efficient advancement of digestive system organoids. Novel techniques, including organ-on-a-chip systems, 3D printing, 4D printing, and others, enable the swift creation of intricate organoids with enhanced efficiency and accuracy, thus compensating for the drawbacks of conventional methods (Hockney et al., 2023).

##### 2.2.2.1 Organ-on-a-chip technology

The technology of organ-on-a-chip employs microfluidic chips to create an organ-like physiological microenvironment. This environment includes various living cells, functional tissue interfaces, biological fluids, and mechanical force stimulation, ultimately forming a model that mimics human physiological or pathological tissues and organs (Deng et al., 2023; Li et al., 2023b; Nasiri et al., 2024). By combining biomaterials, microfluidics, and tissue engineering, this cutting-edge method allows for the precise control of numerous system parameters. It also enables real-time observation of different functional indicators related to tissue and organ activities, showing substantial promise in applications such as organoid development, drug testing, and personalized precision medicine (Palasantzas et al., 2023; Baptista et al., 2024). Organ-on-a-chip organoids can accurately replicate the anatomical structure and physiological/pathological states of tissues/organs, positioning this as a promising culture technology (Shoji et al., 2023).

Wu et al. developed a novel taste bud organoid using organ-on-a-chip technology, which accurately mimics *in vitro* biological taste responses and continues to express key taste receptors even after the third passage, demonstrating high stability and reproducibility (Wu et al., 2023a). This model can be applied to food quality control, disease modeling, and drug screening research. Lee et al. established a gastric organoid chip platform for investigating gastric physiology, disease mechanisms, and drug screening (Lee et al., 2018b). Cherne et al. integrated human dendritic cells and gastric epithelial cells into a microfluidic chip as organoids, creating the first real-time immune-epithelial interaction gastric organoid platform (Cherne et al., 2021). Pinho et al. developed a microfluidic system for the cultivation and expansion of patient-derived colorectal cancer organoids. These organoids demonstrate strong activity and consistent proliferation, making them ideal for disease modeling and drug testing (Pinho et al., 2021). Fang et al. presented a technique that replicates peristalsis in human colonic tumor organoids using a microfluidic platform. This was achieved by integrating lateral micropores and surrounding pressure channels, which generate periodic contractions mimicking intestinal muscle motions (Fang et al., 2021). This system allows precise control over peristalsis amplitude and rhythm, enabling high-throughput organoid culture and providing a more reliable and representative approach for organoid model development. Microfluidic cell culture technology has emerged as an alternative to traditional animal and cell culture models in cancer research (Sontheimer-Phelps et al., 2019). The actions of cancer cells within the microfluidic component of the tumor organoid chip show a significant level of physiological resemblance to *in vivo* environments. This similarity enables the co-culture of various cell types and permits accurate regulation of the physical, mechanical, and biochemical properties of the model, thus realizing a smooth combination of organoid modeling with microfluidic technology (Saorin et al., 2023). Du et al. employed bile duct epithelial cells and integrated organ-chip technology to develop organoids that mimic the bile duct, featuring tubular architectures and barrier capabilities (Du et al., 2020). This novel organ model offers a reliable *in vitro* system for investigating biliary pathophysiology, allowing separate access to the apical and basolateral surfaces of bile duct epithelial cell channels. In 2024, their research progressed further as they introduced vascular components into bile duct-like structures through organ-on-a-chip technology (Du et al., 2023). Meanwhile, Lee et al. described the co-culture of pancreatic cancer cells with pancreatic stellate cells using microfluidic chip methods, thereby creating an early-stage, simplified organ-chip model of pancreatic cancer (Lee et al., 2018a). Subsequently, Bradney et al. embedded the pancreatic cancer cell line KPC from an animal model of spontaneous pancreatic tumorigenesis in Matrigel and placed it in a biochip, thereby constructing an initial pancreatic cancer microenvironment organ-chip model (Bradney et al., 2020). Microfluidic chip technology combines mechanical and biochemical external factors to accurately control local fluid flow, providing potential applications for building organoids of the digestive system (Haque et al., 2021).

##### 2.2.2.2 3D/4D printing technology

3D printing involves the utilization of computer-aided design to fabricate biocompatible materials, cells, and biomolecules into intricate bioactive tissue or organ structures (Kantaros, 2022). This technology boasts several advantages, including cost-effectiveness, high material utilization, a streamlined process, and customization capabilities for organoids. It is characterized by its high degree of personalization, freedom, and precision (Assad et al., 2023; Jing et al., 2023). While 3D printing excels in creating static structures, it falls short in simulating the dynamic behavior of natural tissues and organs (Mandal and Chatterjee, 2024). In comparison, 4D printing expands on 3D printing by adding time as the fourth dimension. This enables the use of stimuli to trigger dynamic transformations in printed structures, leading to a condition of dynamic balance (Kalogeropoulou et al., 2024; Wan et al., 2024). 4D printing is capable of creating highly intricate biological architectures, successfully overcoming certain constraints of 3D printing, and has the potential to transform the fields of tissue engineering and regenerative medicine.

Lee et al. employed 3D printing techniques to fabricate hepatic organoids by using an acellular extracellular matrix (ECM) sourced from liver tissue, together with vessel and biliary structures that closely mimic the native vascular and biliary systems. This innovative model not only showcases bile duct functionality but also displays liver-specific gene expression patterns, highlighting its potential as a valuable tool for *in vitro* drug testing (Lee et al., 2019). As 3D printing technology continues to evolve rapidly, the creation of highly intricate 3D models allows for a more precise representation of the structural and functional characteristics of bile duct-related organs. Additionally, bioprinting technology provides a new foundation for organoid construction, facilitating the progressive reduction in dependence on complex and varied extracellular matrices, thereby enhancing experimental efficiency and outcomes (Kozlowski et al., 2021).

4D printing offers innovative possibilities for creating digestive system organoids capable of changing shape and adjusting functionality in reaction to external factors like temperature, pH levels, or humidity fluctuations. This capability significantly improves their physiological accuracy. Through the use of intelligent, stimuli-responsive materials, 4D printing not only mimics the development and healing mechanisms of the digestive tract but also establishes a foundation for scientists to examine cellular reactions in diverse environments. Consequently, this approach facilitates the creation of more authentic biological response models (Li et al., 2024; Chadwick et al., 2020). However, 4D printing imposes stringent requirements on materials. These smart materials must exhibit precise responsiveness, and high-precision printing technology is crucial for maintaining microstructural consistency. Currently, the fabrication of complex and dynamically responsive digestive system organoids remains technologically and materially challenging.

## 3 Mechanisms of growth and development

The development of organisms is a highly intricate process. Despite advancements in two-dimensional culture techniques and animal models, these methods cannot fully overcome the inherent limitations posed by *in vitro* and *in vivo* discrepancies as well as interspecies differences. Organoid models, however, have demonstrated the ability to recapitulate organismal developmental patterns *in vitro* (Bassi et al., 2021), offering enhanced opportunities to study the mechanisms underlying organogenesis. In 2019, Rosowski et al. successfully simulated early human tooth formation and mesenchymal condensation *in vitro* using scaffold-free cultures of human dental pulp mesenchymal stem cells (Rosowski et al., 2019). During this process, the expression levels of TGF-β1, TGF-β2, and TGF-β3 were upregulated, while the expression of the TGF-β inhibitor Smurf2 was downregulated. Additionally, the expressions of INHBA and its receptor ACVR1 were also upregulated. These findings suggest a signaling transition from BMP to TGF-β during condensation, primarily mediated by Smad2/Smad3. Furthermore, the Notch pathway exhibited increased expression of JAG1 and NOTCH3 receptors, coupled with decreased levels of the inhibitory co-factors histone deacetylase (HDAC) 7 and HDAC11, and an elevated level of FURIN, indicative of autocrine activation. Conversely, the reduced expression of LIMK2 and CYR61 suggests diminished RhoA signaling.

In 2022, Hemeryck et al. developed a dental organoid through the three-dimensional culture of the third molar tooth sac (Hemeryck et al., 2022). They showed that the existence of dental mesenchymal cells, particularly dental pulp stem cells, promoted the differentiation of epithelial stem cells into ameloblasts. Furthermore, they observed that transient elevation of epidermal growth factor promoted the migration of mesenchymal cells to repair injured teeth, underscoring the critical role of mesenchyma-epithelial interactions in tooth development and ameloblast differentiation. Additionally, they found that TGF-β significantly enhanced the simulated enamel formation in dental organoids. In studies on submandibular gland organoids, Nagle et al. reported that these organoids formed branching and lobular structures in a 3D culture system, containing stem cells and other cell types derived from tissues (Nagle et al., 2016). Serrano et al. discovered that parotid stem cells could extend and expand *in vitro*, forming lobular structures with differentiation potential in parotid organoids (Serrano Martinez et al., 2021). Their findings indicated that Wnt signaling is widely recognized as a key driver for organoid formation by various adult epithelial cells. Activation of Wnt signaling promotes postnatal development of salivary glands and tissue regeneration following duct ligation, playing a crucial role in maintaining and expanding stem cells and organoids in both parotid and submandibular glands (Serrano Martinez et al., 2021). Collectively, organoid models are anticipated to become an essential tool in biomedical research, offering novel insights and methodologies for studying organ growth and development mechanisms.

As an emerging *in vitro* model, gastrointestinal (GI) organoids are increasingly utilized to investigate the mechanisms underlying the growth and development of the gastrointestinal tract. These organoids, derived from pluripotent stem cells, exhibit the ability to recapitulate the structural and functional characteristics of the *in vivo* gastrointestinal tract (Poling et al., 2024). Through the use of GI organoids, researchers can reconstruct the developmental processes of the GI tract *in vitro* and elucidate the associated molecular mechanisms. Culturing GI organoids *in vitro* enables the observation of complex physiological events, such as endoderm formation, intestinal tube morphogenesis, and villus development (Ghorbaninejad et al., 2023; Singh et al., 2020). Villus formation represents a highly intricate patterning process that involves dynamic interactions between epithelial and mesenchymal cells. Huycke et al. employed time-lapse imaging technology to visualize the processes of interface folding and aggregate formation, thereby revealing the initiation and progression of small intestinal villus development (Huycke et al., 2024). Furthermore, when combined with gene-editing technologies, GI organoids provide a powerful platform for studying the roles of specific genes in gastrointestinal development. For instance, Zhao et al. demonstrated that knocking out the *Znhit1* gene in mouse intestinal epithelial cells impaired the maintenance of intestinal stem cells, consequently disrupting postnatal intestinal homeostasis establishment and affecting overall intestinal development (Zhao et al., 2019a). Additionally, Hamilton et al. reported that esophageal organoids overexpressing *ASCL2* exhibited increased basal markers (p63), decreased suprabasal markers (Krt13, Wnt5a), and reduced stem cell markers (NT5E). This suggests that ASCL2 overexpression modulates organoid differentiation and proliferation, playing a critical role in coordinating the fate decisions of esophageal epithelial cells (Hamilton et al., 2022).

The study of pancreatic biology has been constrained by the absence of an adequate *in vitro* model to elucidate the mechanisms governing pancreatic growth and development. Advancements in technology have enabled the creation of 3D culture systems, referred to as organoids, which can be developed from either primary cells or reprogrammed stem and progenitor cells. Due to their ability to self-organize into functional structures that replicate the intricacy and function of natural tissues, these organoids have become powerful tools for studying pancreatic growth, development, and associated diseases. Andersson-Rolf et al. developed a highly stable human fetal pancreatic organoid (hfPOs) system through embedding culture technology utilizing 15 to 16 gestational weeks (GW) of human fetal pancreatic tissue (Andersson-Rolf et al., 2024). This system replicates the natural epithelial complexity of the human fetal pancreas. In a living organism, lobulation begins approximately at 14 weeks, followed by the emergence of acinar cells containing zymogen granules. Before reaching the 12- to 14-week stage, the pancreas is primarily made up of undifferentiated cells arranged in tubular structures. Furthermore, the researchers detected the expression of various digestive enzymes produced by the acinar cells of hfPOs, such as trypsinogen (PRSS1 and PRSS2), proteases (CTRB1, CTRB2, and CTRC), and elastases (CELA2A and CELA3A/B). This model holds significant promise for studies on human pancreatic development, physiology, disease mechanisms, and regenerative medicine. Cherubini et al. constructed a tissue-derived human pancreatic organoid with robust stability using embedding culture techniques (Cherubini et al., 2024). They confirmed the heterogeneity of functional pancreatic duct subsets and demonstrated that pancreatic organoids follow a precise developmental trajectory, utilizing multiple signaling pathways, including EGF and SPP1, to facilitate cell-to-cell communication and maturation. This lays a robust groundwork for upcoming *in vitro* diagnostics and translational research focused on pancreatic health and disease. Fernandez et al. developed pancreatic organoids and pinpointed ductal cell populations that exhibit strong organoid-forming capabilities along with the potential to differentiate into endocrine and exocrine cells in a laboratory setting. These populations include Wnt-responsive cells, ciliated cells, and Flrt3-positive cells. The researchers further examined the organoid-forming capacity and endocrine differentiation potential of these cell populations, shedding light on their possible contributions to pancreatic regeneration (Fernández et al., 2024).

The development of the digestive system is a highly regulated process involving the synergistic action of multiple signaling pathways. For example, the BMP (bone morphogenetic protein) signaling pathway plays a crucial role in the morphogenesis of the digestive system (Zhang and Que, 2020). Studies in animal models, tissue organoids, and human pluripotent stem cells have significantly expanded our understanding of the role of BMPS in GI organ development and homeostasis. Notch signaling pathway also plays an important role in digestive tract tumors, and reasonable regulation of Notch signaling pathway may have an impact on the occurrence and development of tumors (Liu et al., 2024). In addition, Wnt signaling pathway also plays a key role in the development of digestive system, especially in the occurrence and development of colorectal cancer (Zhang et al., 2024). Digestive system organoids provide unprecedented opportunities to study the development, physiological functions, and diseases of the digestive system. A deep understanding of the growth and development mechanisms of organoids will help to develop more effective disease treatment strategies and provide new ideas for regenerative medicine.

## 4 Disease modeling and mechanism studies

### 4.1 Oral organoids

The modeling of disease organoids requires a relatively short period, allowing for more intuitive tracking and investigation of tissue and cellular responses and changes. This approach holds significant promise in disease modeling and mechanism research. A few countries have established organoid biobanks for cancer, confirming the feasibility of using organoids as experimental models for targeted therapy. In an oral squamous cell carcinoma (OSCC) organoid model, Zhao et al. demonstrated that co-culturing cancer-associated fibroblasts (CAFs) with CD44-expressing cancer stem cells (hereafter referred to as CD44^+^ cells) resulted in the formation of OSCC organoids (Zhao et al., 2021). They observed increased expression levels of CD44 and OCT-4 in these organoids through immunofluorescence and Western blot analyses, indicating that CAFs enhance the organoid-forming capability of CD44^+^ cells. In 2023, researchers further discovered that CAFs in OSCC organoids express nicotinamide N-methyltransferase, which reduces the enrichment of H3K27me3 at the promoter region of the lysyl oxidase gene. This reduction leads to increased deposition of type I collagen, thereby promoting the growth and development of OSCC (Zhao et al., 2023).

Zhao et al. identified a therapeutic target for OSCC (Zhao et al., 2019b). By silencing monocarboxylate transporter 1 (MCT1), the levels of lactate, which is associated with tumor prognosis, were reduced, and the proliferative capacity of cancer cells was diminished. Therefore, inhibiting MCT1 can serve as a potential therapeutic target for OSCC treatment. Carcinoembryonic antigen-related cell adhesion molecule 1 (CEACAM1) binds to CEACAM1 on natural killer cells and Tim3 on T cells, thereby suppressing the body’s anti-tumor immune response. Blocking CEACAM1 using targeted antibodies or small molecules may restore the body’s anti-tumor immunity and represents a promising new immunotherapy approach for head and neck squamous cell carcinoma (HNSCC) (Tsang et al., 2022). Considering the individual variability of tumors, Driehuis et al. established HNSCC organoids from 31 patients *in vitro* and observed diverse responses to cisplatin, carboplatin, cetuximab, and radiotherapy (Driehuis et al., 2020a). The *in vitro* responses mirrored the clinical outcomes of patients, highlighting the potential of tumor-derived organoids to guide personalized therapies.

### 4.2 Esophageal organoids

As an effective tool for modeling the structure and function of the esophagus, esophageal organoids have gained widespread application in recent years, particularly in the study of esophageal inflammation and esophageal cancer. Compared with PSCs-derived organoids, tissue-derived esophageal organoids are more straightforward to construct and better preserve certain characteristics of the original tissue. Consequently, tissue-derived esophageal organoids play a pivotal role in the study of esophageal disease pathogenesis and their applications in regenerative medicine (Cabeza-Segura et al., 2023). Nakagawa et al. developed an organoid model of eosinophilic esophagitis using patient-derived esophageal tissues (Nakagawa et al., 2020). Research has demonstrated that eosinophilic esophagitis induces basal cell proliferation, and exogenous recombinant cytokines such as IL-13 can prompt organoids to replicate the inflammatory response characteristic of this condition. This study underscores the potential of the eosinophilic esophagitis organoid model to simulate disease pathogenesis through induced inflammatory responses, thereby facilitating the identification and development of potential therapeutic strategies. Advances in tumor-derived organoid culture techniques have led to the successful establishment of several esophageal cancer models. Organoids derived from tumor tissues exhibit high similarity to primary tumors and preserve their heterogeneity, providing a platform for personalized treatment options for cancer patients. Esophageal squamous cell carcinoma (ESCC), which is the primary subtype of esophageal cancer in Asia, represents 40% of worldwide esophageal cancer cases (Thrift, 2021). Kijima et al. developed a technique for cultivating ESCC organoids derived from patients. These organoids can be efficiently produced from single-cell suspensions embedded in a basement membrane matrix within 2 weeks, with a success rate of around 60%. They also investigated the *ex vivo* response of these organoids to 5-fluorouracil, revealing that cancer cells with high CD44 expression may contribute to tumor resistance (Kijima et al., 2019).

Barrett’s esophagus (BE) is recognized as a precancerous lesion associated with esophageal adenocarcinoma (EAC), a type of cancer with a poor prognosis and rapidly increasing incidence in Western countries (Peters et al., 2019). In 2011, Sato et al. pioneered the generation of an esophageal epithelial organoid using biopsy tissue from BE, marking the inception of organoid-based research for this condition (Sato et al., 2011). The cellular origin of esophageal tumors remains a subject of debate, and existing studies have not conclusively determined whether esophageal adenocarcinoma (EAC) develops from BE, as approximately half of EAC patients do not exhibit BE metaplasia at diagnosis. In 2021, Nowicki-Osuch and colleagues leveraged esophageal epithelial organoids to show that BE emerges from the gastric cardia and is propelled by c-MYC and hepatocyte nuclear factor 4 alpha (HNF4α). This discovery suggests that EAC develops via BE-like epithelial metaplasia, filling a crucial gap in prior research and highlighting the significance of esophageal organoids in modeling the esophagus (Nowicki-Osuch et al., 2021). Kunze and collaborators explored the connection between Notch signaling and goblet cells in BE, demonstrating that activation of the Notch pathway results in decreased goblet cell density in BE, which is closely linked to the activation of nuclear factor kappa-B (Kunze et al., 2020). Considering the pivotal role of Notch signaling in tumor formation, these insights offer meaningful contributions to future EAC prevention strategies. The combination of gene editing with organoid technology has expanded the utility of organoids in elucidating disease mechanisms. Liu and associates utilized CRISPR/Cas9 technology to examine the function of the Wnt signaling pathway in tumor transformation associated with BE (Liu et al., 2018). Their results indicated that activating the Wnt signaling pathway enhances proliferation and replication capabilities while reducing apoptosis in BE organoids compared to their wild-type counterparts. At present, esophageal organoids are primarily applied in esophageal cancer research, with limited exploration in other diseases. The unclear cellular origin of esophageal tumors has led most studies to focus on elucidating the mechanisms of tumorigenesis, which may explain the restricted use of esophageal organoids in researching other conditions. A large number of studies have shown the substantial importance of esophageal organoids in simulating tumor progression and performing tumor drug testing. Looking ahead, esophageal organoids hold promise for expanding into the study of other esophageal diseases.

### 4.3 Gastric organoids

Rodents and gastric cancer cell lines are frequently utilized models for investigating *Helicobacter pylori* infection; however, both models possess inherent limitations. Mouse models typically exhibit only mild inflammation and do not progress to gastric ulcers or gastric cancer (Idowu et al., 2022). Gastric cancer cell lines often harbor mutated oncogenes and lack the capacity for self-renewal (Idowu et al., 2022). In contrast, gastric organoids can faithfully replicate the structural complexity of the stomach, thereby playing a crucial role in elucidating *H. pylori* infection and gastric cancer pathogenesis.

McCracken et al. directly microinjected *H. pylori* into the epithelial lumen of organoids, observing the resultant pathophysiological responses (McCracken et al., 2014). This study demonstrated that cytotoxin-associated gene A could invade organoid epithelial cells and interact with the c-Met receptor, underscoring its significance in *H. pylori* infection. Gastrointestinal pancreatic neuroendocrine neoplasms (GEP-NEN) represent a rare disease, characterized by limited clinical samples, which has historically hindered research progress. Organoids offer a promising solution to this challenge. Kawasaki et al. established a library of 25 GEP-NEN organoids derived from patient gastric tissues and conducted comprehensive analyses, including whole-genome sequencing (Kawasaki et al., 2020). Their findings revealed frequent *RB1* mutations and extensive chromosomal aberrations, which closely resemble the genetic alterations observed in adenocarcinoma organoids. Additionally, CRISPR-Cas9 technology was employed to knockout *TP53* and *RB1* genes in normal gastric organoids, generating a model that accurately reflects the genetic profile of GEP-NEN for mechanistic studies (Kawasaki et al., 2020). Collectively, gastric organoids provide a more effective platform for studying gastric diseases and will likely become an indispensable tool in this field.

### 4.4 Small intestinal organoids

Small intestinal organoids are capable of self-assembling into micro-organs with intricate three-dimensional architectures, encompassing a diverse range of intrinsic intestinal cell types, including intestinal epithelial cells, goblet cells, and Paneth cells (Ghorbaninejad et al., 2023). This high level of structural and functional fidelity allows small intestinal organoids to more accurately recapitulate the *in vivo* physiological state of the intestine, thereby providing a robust model for elucidating the mechanisms underlying intestinal diseases.

The small intestinal organoid system, established as the earliest organoid model, has been employed to study a range of diseases, such as cystic fibrosis and infections caused by bacteria and viruses. Cystic fibrosis is a rare genetic condition marked by mutations in the cystic fibrosis transmembrane conductance regulator (CFTR) chloride channel within epithelial cells (Yin et al., 2019). Reproducing the varied phenotypes of CFTR mutants poses significant challenges for traditional cell lines and animal models, and there is still a lack of effective clinical therapies. As a result, organoids have become an essential tool for researching these disorders.

Dekkers et al. introduced a novel method termed “forskolin-induced swelling (FIS)” for the functional assessment of cystic fibrosis using small intestinal organoids (Dekkers et al., 2013). This study demonstrated that forskolin activates CFTR in organoids, resulting in observable swelling. The extent of this swelling is diminished in samples lacking functional CFTR or harboring CFTR mutations. FIS has established a robust research model for drug screening in cystic fibrosis and offers potential for personalized therapeutic approaches. Small intestinal organoids exhibit characteristics closely resembling those of human intestinal epithelium, making them an ideal platform for investigating the pathogenesis and treatment of infectious diseases. Norovirus, an enterovirus responsible for acute gastroenteritis, lacks an effective antiviral drug or vaccine due to the absence of a suitable *in vitro* culture system (Flynn et al., 2024). While traditional laboratory methods for detecting norovirus RNA are highly sensitive, they cannot differentiate between infectious and non-infectious viral particles. Chan et al. successfully cultured norovirus in intestinal organoids and utilized real-time reverse transcription PCR to determine the threshold of norovirus replication (Chan et al., 2019). They found that when the C t value was ≤30, the virus replicated efficiently within organoids, providing a valuable tool for assessing viral infectivity in clinical settings. Additionally, rotavirus, *Shigella*, and *Escherichia coli*, which are major pathogens causing diarrhea, have also been studied using organoid models.

Finkbeiner et al. demonstrated that small intestinal organoids are susceptible to infection by both experimental rotavirus (simian SA11) and clinical rotavirus isolates (Finkbeiner et al., 2012). Furthermore, the study revealed that iPSC-derived small intestinal organoids support pathogen replication, indicating their potential for culturing intestinal pathogens that are challenging or impossible to grow using traditional models. Pradhan and colleagues developed a model using Shiga toxin-infected small intestinal organoids to examine how small intestinal tissues respond biologically to Shiga toxin exposure (Pradhan et al., 2020). Their study revealed that Shiga toxin triggers necrosis and apoptosis in both intestinal epithelial and stromal cells. Additionally, preserving the integrity of the intestinal epithelial barrier strengthens the organoids’ resilience against Shiga toxin infection. Barron et al. used non-pathogenic *E. coli* ECOR2 to microinject small intestinal organoids and discovered that deletion of the *RpoS* gene reduces ECOR2’s ability to colonize these organoids (Barron et al., 2020). Serra et al. identified yes-associated protein 1 (Yap1) as a signaling factor that detects organoid integrity; upon organoid disintegration, Yap1 activation drives tissue repair, which subsequently induces specific Yap1 activation in local cell clusters (Serra et al., 2019). Yap1 also promotes delta-like canonical Notch ligand 1 expression and Paneth cell formation *in vivo*. The Wnt signaling pathway plays a crucial role in organoid culture. Miao et al. engineered a modified Wnt molecule that forms heterodimers with Wnt Frizzled receptors (Fzd) and LDL receptor-related protein 6 (Miao et al., 2020). Administration of Fzd-specific Wnt agonists enhances the proliferation of adult intestinal crypt cells and improves the long-term proliferation and maintenance of organoids. In summary, organoids hold significant potential for modeling diseases and investigating disease mechanisms in the small intestine. In addition, the enteric nervous system (ENS) plays a crucial role in the regulation of intestinal function. The co-culture of enteric nerves and intestinal organoids can mimic the interaction between enteric nerves and the epithelium, thereby offering a novel model for investigating ENS function and associated diseases (Özkan et al., 2024). A sophisticated 3D culture technique was developed to enable the co-culture of small intestinal organoids with myenteric and submucosal neurons. Through the refinement of isolation methods, intestinal organoids containing both intestinal neurons and glial cells from the two nerve plexuses were successfully established, providing a unique platform for studying the regulatory mechanisms of the enteric nervous system.

### 4.5 Liver organoids

In terms of organ development, homeostasis maintenance, and pathogenesis, organoid models are more accurate than animal models in providing basic information similar to that of the human body. So far, researchers have successfully constructed different kinds of liver disease models.

#### 4.5.1 Liver cancer

Liver cancer primarily encompasses both primary and secondary types. Primary liver cancer originates from the liver tissue itself and can be categorized into three main types based on histological characteristics: hepatocellular carcinoma, intrahepatic cholangiocarcinoma, and the less common mixed liver cancer. Most cases of liver cancer are diagnosed in the middle to late stages, leading to a poor prognosis. Consequently, early diagnosis, prevention strategies, and standardized treatment protocols for liver cancer are of paramount importance. Liver cancer organoids serve as an excellent model for investigating the molecular mechanisms underlying the development of malignant liver tumors and play a crucial role in identifying therapeutic targets and screening potential drugs (Ji et al., 2023).

Yang et al. employed the organoid culture technique to successfully expand fetal liver-derived hepatocytes by stimulating the Hippo-YAP signaling pathway, leading to the malignant transformation of fetal hepatocyte organoids into tumor structures that resemble fetal hepatoblastoma (Yang et al., 2022). In a separate study, Khedr et al. established a hepatocellular carcinoma (HCC) organoid model using embedding culture methods in combination with human bone marrow-derived mesenchymal stem cells. This model was utilized to investigate the function of HIF-1A within the tumor microenvironment. The findings indicated that four HIF-1A downstream target genes—*HK2*, *ENO2*, *PFKFB3*, and *SLC2A1*—are implicated in metabolic processes and could potentially serve as therapeutic targets for HCC (Khedr et al., 2024).

#### 4.5.2 Cirrhosis and liver fibrosis

Hepatic fibrosis represents a critical phase in the progression of chronic liver disease, characterized by the abnormal accumulation and excessive deposition of extracellular matrix within the liver due to repeated exposure to various stimuli (Pei et al., 2023). The advancement of hepatic fibrosis can culminate in cirrhosis, marked by nodule formation and pseudolobular structures, ultimately leading to the disruption of normal liver architecture and blood supply (Jangra et al., 2022). Histologically, liver fibrosis is reversible if aggressively treated during this stage. However, once it progresses to cirrhosis, reversal becomes exceedingly difficult, often resulting in poor prognosis and high mortality rates. The etiology of both conditions is largely similar, encompassing viral hepatitis, excessive alcohol consumption, immune and circulatory disorders, prolonged exposure to drugs, chemicals, and toxins, cholestasis, parasitic infections, genetic and metabolic diseases, and malnutrition (Friedman and Pinzani, 2022).

Ouchi et al. introduced free fatty acids into liver organoids for cultivation. As the concentration of free fatty acids increased, the organoids exhibited progressive inflammation and fibrosis (Ouchi et al., 2019). Additionally, they discovered that FXR agonist-mediated inhibition of reactive oxygen species mitigated steatohepatitis, offering a novel approach to explore personalized treatment strategies for inflammation and fibrosis in humans.

#### 4.5.3 Fatty liver

Fatty liver represents a heterogeneous group of conditions characterized by the interaction of genetic predisposition, environmental factors, and metabolic stress, resulting in excessive lipid accumulation within hepatocytes. This condition constitutes a common hepatic pathological change rather than an independent disease entity. It encompasses alcoholic fatty liver disease, non-alcoholic fatty liver disease (NAFLD), including non-alcoholic steatohepatitis (NASH), and other specific types, with NASH being the most prevalent form. Fatty liver disease is reversible; early detection and intervention can control its progression or even restore normal liver function. However, if left unchecked, it may lead to structural alterations in the liver, progressing to hepatitis, fibrosis, cirrhosis, and potentially hepatocellular carcinoma. Given the escalating global obesity rates, the prevalence of fatty liver disease is expected to rise significantly over the coming decades, imposing substantial burdens on both societal and individual health (Lazarus et al., 2023).

McCamon et al. successfully developed a liver organoid model using biopsy specimens from NASH patients, which accurately mimics the pathophysiological state of NASH-affected livers. Utilizing single-cell RNA sequencing technology, they classified and phenocopied various cell subsets within NASH liver tissues, elucidating cellular state changes during disease progression (McCarron et al., 2021). Comparative metabolic analyses between NASH and healthy liver tissues revealed that NASH tissues exhibit lipid overload and oxidative stress.

#### 4.5.4 Viral hepatitis

Viral hepatitis, classified as a Group B infectious disease, is primarily caused by various types of hepatitis viruses. In some cases, patients may develop chronic conditions that can progress to liver cirrhosis and pose a risk of malignant transformation. Viral hepatitis is prevalent globally, including in the United States, where hepatitis B virus (HBV) is the predominant cause of chronic hepatitis, cirrhosis, and hepatocellular carcinoma (Nevola et al., 2023). Consequently, it is imperative to establish an organoid model for HBV infection and investigate novel therapeutic strategies for managing chronic HBV infection (Guo et al., 2023).

Future research by De Crignis et al. aims to cultivate liver organoids from healthy donor liver tissue and subsequently infect them with recombinant viruses or HBV to generate HBV-infected organoids. This model has demonstrated the ability to generate covalently closed circular DNA, as well as express HBV early antigen, intracellular HBV RNA and proteins. Additionally, it can produce infectious HBV particles (De Crignis et al., 2021).

### 4.6 Biliary organoids

Biliary organoids provide a crucial platform for studying diseases like biliary atresia, biliary tract cancer, and primary sclerosing cholangitis, enabling a deeper understanding of the underlying disease mechanisms. Chen et al. were the first to develop a method for cultivating biliary organoids using gel embedding. These organoids were then co-cultured with rotavirus, allowing for the successful creation of a biliary atresia (BA) disease model (Chen et al., 2020). Their findings demonstrated that rotavirus causes damage to biliary tract cells through interactions with host cells, which contributes to the onset of BA. Additionally, they suggested that suppressing rotavirus replication and providing antibodies targeting the VP7 protein of rotavirus might serve as promising treatment approaches for BA. Maier et al. reported a protocol for the establishment of cholangiocarcinoma organoids in stable culture conditions. They mechanically dissociated cholangiocarcinoma tissues and enzymatically digested them with tissue-specific enzymes for 2 h, followed by filtration through a 40–100 μm cell strainer and differential centrifugation at 200 g for 3 min. The isolated cells and cell aggregates were subsequently co-seeded in a matrix gel supplemented with ROCK inhibitor, forskolin, insulin, transferrin, and selenite to form stable cholangiocarcinoma organoids (Maier et al., 2021). Du et al. utilized organ-chip technology to create a vascularized bile duct chip-based organoid model derived from PSC) (Du et al., 2023). The expression patterns of critical markers, including bile duct cell indicators, polarity proteins, collagen IV, laminin, bile salt transporters, secretin receptors, and tight junction proteins (such as zonula occludens-1), closely matched those found in primary bile duct cells obtained from PSC patients. This sophisticated disease model provides substantial benefits for exploring the physiological and pathological processes associated with PSC. In recent years, Jalan-Sakrikar et al. successfully reprogrammed fibroblasts from PSC patients into iPSCs and cultivated them under three-dimensional conditions to establish PSC organoids (Jalan-Sakrikar et al., 2022).

Existing biliary tract models, including two-dimensional cell cultures, are inadequate for replicating the complex structure of the biliary system. Moreover, these models present challenges in precisely controlling the dimensions of the biliary tract and the positioning of cells. Consequently, there is a critical need for an advanced *in vitro* biliary tract model to facilitate comprehensive studies of biliary physiology and pathology. Organoids, which are distinguished by their distinctive spatial structure and cell-specific properties, hold promise for tissue regeneration and the recovery of many original organ functions. This feature renders them a perfect model for exploring the physiological and pathological processes of the biliary tract. Jalan-Sakrikar et al. successfully reprogrammed fibroblasts derived from PSC patients into hiPSCs and then generated biliary organoids through a three-dimensional culture method (Jalan-Sakrikar et al., 2022). Through electron microscopy, they observed that these organoids were diminutive, lacked a central lumen, and exhibited accelerated aging. Additionally, they noted increased secretion of fibronectin, interleukin-6, and C-C motif chemokine ligand 2, which highlighted the disease-specific characteristics of PSC. Amarachintha et al. generated bile duct atresia cystic organoids (BACOs) by culturing liver tissue from infants with biliary atresia in a three-dimensional environment (Amarachintha et al., 2022). Transmission electron microscopy showed a limited number of ciliated cells with abnormal lateral cilia development, which may be associated with decreased levels of F-actin, β-catenin, and ezrin secretion. In a separate experiment, it was observed that BACOs had reduced expression of the tight junction protein zonula occludens 1 in biliary epithelial cells, resulting in impaired barrier function and elevated permeability. Additionally, stimulation of the EGF/FGF signaling pathway in biliary epithelial cells promoted epithelial differentiation and enhanced the integrity of the biliary epithelial barrier (Amarachintha et al., 2022). Verstegen et al. developed a cystic fibrosis model using organoids that exhibited normal chloride channel and MDR1 transporter activity but lacked functional CFTR channel activity (Verstegen et al., 2020).These studies highlight the crucial role of biliary organoids as a platform for visualizing and studying metabolic and regulatory processes within the biliary system.

### 4.7 Pancreatic organoids

Advancements in pancreatic organoid technology have enabled the development of three-dimensional models that accurately replicate the heterogeneity, structure, and function of native pancreatic tissue, which is crucial for modeling pancreatic diseases (Liu et al., 2023). Pancreatic organoids can emulate a diverse array of pancreatic cell types, including mature ductal cells and acinar cells. These 3D models facilitate a more profound understanding of drug mechanisms of action, offer faster and more cost-effective assessments, reduce reliance on animal models, and enhance the prediction of patient responses.

#### 4.7.1 Pancreatic cancer

Advancements in pancreatic organoid technology have demonstrated their capability to faithfully replicate ductal pancreatic cancer characteristics observed in both human and murine models. Through the utilization of organoid models, researchers can identify and compare tumor alterations with normal tissues, which is crucial for elucidating the distinct features of pancreatic cancer (Below et al., 2022).

Moreira et al. employed RNA sequencing and mass spectrometry to analyze gene expression and proteomics in three-dimensional mouse pancreatic organoids, revealing that these molecular profiles are indicative of tumor progression (Moreira et al., 2018). Bailey et al., through an integrative analysis combining whole-genome, exome, and RNA sequencing data from 456 pancreatic cancers, delineated four distinct subtypes of pancreatic ductal adenocarcinoma: squamous cell carcinoma, pancreatic progenitor-like tumors, immunogenic tumors, and aberrantly differentiated exocrine tumors. Each subtype was associated with specific molecular pathways, histopathological characteristics, and prognostic implications, providing valuable insights for the development of targeted therapies (Bailey et al., 2016). By 2025, Tabe and colleagues established a co-culture system combining patient-derived pancreatic ductal adenocarcinoma (PDAC) cells with hiPSC-derived mesenchymal and endothelial cells. This approach led to the creation of a PDAC organoid model referred to as the Fused Pancreatic Cancer Organoid (FPCO) (Tabe et al., 2025). Additionally, they integrated macrophages derived from the THP-1 cell line into the FPCO system. These macrophages function as a source of tumor-associated macrophages (TAMs), which represent a key element of the tumor microenvironment (TME), thereby generating the M0-FPCO model. This approach effectively recapitulates the heterogeneity of TAMs within PDAC organoids, elucidating their role in endothelial network formation and modulation of PDAC cell properties. Sada et al. demonstrated that a humanized anti-CKAP4 antibody (Hv1Lt1) inhibited pancreatic cancer progression by blocking the DK1-CKAP4 pathway and reducing AKT activity. Notably, Hv1Lt1 promoted significant infiltration of cytotoxic T cells into the tumor microenvironment (Sada et al., 2024). Moreover, the combination of Hv1Lt1 with other chemotherapeutic agents exhibited enhanced efficacy compared to monotherapy, highlighting its potential as an effective anticancer therapy. Collectively, these studies underscore the utility of pancreatic cancer organoids as a novel platform for investigating pancreatic cancer mechanisms and gene functions.

#### 4.7.2 Diabetes

Diabetes mellitus arises from multifactorial etiologies resulting in impaired glycemic regulation and subsequent multi-organ dysfunction. Type 1 diabetes is characterized by absolute insulin deficiency, while Type 2 diabetes manifests as relative insulin insufficiency. Islet organoids have emerged as a novel research platform with significant potential due to their unique adaptability and long-term viability. These structures differ markedly from pancreatic organoids; the latter primarily consist of ductal epithelial cells for cancer studies, whereas islets with endocrine functions are utilized in β-cell research for diabetes.

In the modeling of diabetes, islet-like cell clusters were generated through *in vitro* culture of hESCs and iPSCs. These clusters demonstrated the ability to respond to glucose stimulation and secrete insulin (Molakandov et al., 2021). Eiji et al. developed a protocol for generating human islet organoids from iPSCs via nonclassical WNT4 signaling. They observed that these organoids could provide glycemic control and evade potential cellular immunity in immunocompetent diabetic mice by overexpressing immune checkpoint proteins, thereby establishing an effective platform for diabetes research (Yoshihara et al., 2020). Moreover, human amniotic epithelial cells (hAEC) are recognized for their ability to regenerate tissue, modulate immune responses, and reduce inflammation (Lebreton et al., 2022). By integrating hAEC into organoid models, there is not only an improvement in blood circulation but also enhanced insulin production, balanced immune reactions, reduced inflammation post-transplantation, and extended survival of islets, thereby increasing the likelihood of successful transplantation (Lebreton et al., 2020). Furthermore, islet organoids provide a platform for exploring the connection between diabetes and various complications, such as the link between NAFLD and type 2 diabetes (Kimura et al., 2022). These organoids are also being combined with cutting-edge technologies like gene chips and 3D bioprinting, allowing scientists to delve deeper into the complexities of diabetes (Yin et al., 2022). As a promising technology, islet organoids hold significant potential for future applications.

In summary, organoids have emerged as a versatile platform for simulating various organs of the digestive system, including the oral cavity, stomach, intestine, liver, and pancreas. They serve as a novel tool for investigating inflammation, tumors, and refractory diseases within the digestive system. Gastric organoids can be employed to study *H. pylori* infection and the pathogenesis of gastric cancer, while intestinal organoids are capable of mimicking the heterogeneity of the intestinal epithelium. Liver organoids facilitate the exploration of the interplay between inflammation and fibrosis, and pancreatic organoids enable the examination of the relationship between genetic and proteomic features and pancreatic tumors. Notably, intestinal organoids can be co-cultured with myenteric and submucosal neurons to form organoids with a rudimentary nervous system, thereby enhancing our understanding of the enteric nervous system. Furthermore, intestinal organoids co-cultured with mesenchymal stem cells, immune cells, and gut microbiota can replicate complex cell-to-cell interactions and host-microbe interactions in the gut, offering new insights into inflammatory bowel diseases and infectious diseases. In conclusion, organoids of the digestive system represent an excellent disease model and provide a powerful tool for elucidating the pathogenesis of digestive system disorders.

## 5 Drug screening

Drug development from preclinical stages to clinical application typically progresses through three key phases: discovery, preclinical research, and clinical trials. Clinical trials are categorized into four phases, each associated with significant time investment and inherent research risks.

### 5.1 Oral organoids

Wang et al. utilized salivary gland organoids to investigate the mechanism of progenitor cells in response to β-blockers for treating salivary insufficiency (Wang et al., 2021). Their findings revealed that β-blockers induce a reduction in Notch signaling within intercalated duct cells, thereby impeding the proliferation and differentiation of these cells into acinar cells, leading to persistent hypopsialsecretion in patients on β-blocker therapy. Tanaka et al. refined the spheroid culture method for tumor cells, demonstrating that regardless of the status of the tumor suppressor gene *TP53* or human papillomavirus, organoids resembling original head and neck tumors can be formed (Tanaka et al., 2018). This model allows for predicting *in vivo* drug sensitivity of tumor cells, indicating its potential for drug screening and toxicity simulation. Belair et al. identified that tributyltin oxide, all-trans retinoic acid, valproic acid, theophylline, and triamcinolone acetonide interfered with palatal fusion among 12 putative teratogens (Belair et al., 2018). Tigani et al. discovered that triethylene glycol dimethacrylate, a component in dental restorations, exhibits toxic effects on gingival and dental pulp tissues, inhibiting cell migration and aggregation, potentially suppressing the expression of adhesion receptors necessary for cell-ECM connections, and altering cellular structure and morphology (Tigani et al., 2019). Driehuis et al. utilized mouse tongue epithelial organoids to demonstrate that acyclovir can inhibit herpes simplex virus 1 proliferation (Driehuis et al., 2020a). Leucovorin serves as an antidote for methotrexate toxicity, mitigating chemotherapy-induced damage, including oral mucositis, when administered within 72 h post-methotrexate treatment (Driehuis et al., 2020b).

### 5.2 Gastric organoids

Gastric cancer is a complex disease characterized by diverse histological features and molecular subtypes. To elucidate the mechanisms underlying its development, it is essential to investigate the specific expression patterns of these features in appropriate models.

Nanki et al. utilized CRISPR/Cas9 technology to generate gastric cancer organoids harboring multiple mutations. They also established a biobank comprising 37 patient-derived gastric cancer organoids, thereby constructing a comprehensive resource for studying genetic and histopathological changes (Nanki et al., 2018). This biobank facilitates modeling, drug screening, and personalized treatment strategies for gastric cancer. Yan et al. established an additional biobank consisting of gastric cancer organoids derived from 34 patients. This collection included almost all recognized molecular subtypes and mutation profiles, following a meticulous sample selection process (Yan et al., 2018). They conducted extensive whole-exome and transcriptome analyses, providing detailed genomic data on tumors. Additionally, they performed large-scale drug screenings, revealing significant sensitivity of tumor organoids to napabucasin, abemaciclib, and ataxia telangiectasia and Rad3-related inhibitors such as VE-822. Chemotherapy remains a primary treatment modality for gastric cancer; however, challenges like drug resistance and adverse reactions persist. Ouyang et al. developed a selective inhibitor of signal transducer and activator of transcription 3 (STAT3), W1131, which was tested in gastric cancer organoids (Ouyang et al., 2022). It was found that W1131 could reduce tumor cell resistance to 5-fluorouracil by inhibiting STAT3 activity. Zou et al. investigated nano-formulations with fewer adverse effects, comparing the efficacy of two paclitaxel nano-formulations in patient-derived gastric cancer organoids (Zou et al., 2022). They observed that both nanoparticles demonstrated anti-tumor effects, but liposomal paclitaxel exhibited superior cytotoxicity compared to albumin-bound paclitaxel. This study highlights the potential of PDOs as an effective platform for evaluating nanomedicine drugs, suggesting that more such agents may be tested using organoid models in the future. In addition, the recent adoption of conditioned medium as an alternative culture method for recombinant hepatocyte growth factors has substantially decreased the cost of culturing human gastrointestinal tract (GIT) organoids. This advancement facilitates large-scale cultivation of GIT organoids and compound screening. Despite existing challenges in GIT organoid development, such as their inability to form paired structures, limited cell type diversity, and reliance on single drug exposure patterns, these organoids hold significant potential for drug screening (Zhou et al., 2024a). The utilization of GIT organoids in this context is anticipated to enhance the precision of medical treatments for patients with gastrointestinal diseases.

### 5.3 Small intestinal organoids

Human intestinal cell lines, such as Caco-2, have traditionally served as foundational platforms for drug development (Bein et al., 2018). The emergence of small intestinal organoids has introduced a novel and advanced research platform for drug screening. Vijftigschild et al. utilized the FIS model to screen small molecule compounds regulated by G protein-coupled receptors, identifying β2-adrenergic receptor agonists as potent inducers of CFTR function (Vijftigschild et al., 2016). This research highlights the promise of small intestinal organoids as a reliable preclinical model for designing and assessing effective treatments for cystic fibrosis. Yin et al. utilized human small intestinal organoids to identify potential antiviral compounds against rotavirus infection, revealing that cyclosporine A and mycophenolic acid significantly hindered rotavirus replication. These findings validated the practicality of employing small intestinal organoids in drug investigations targeting intestinal infections (Yin et al., 2018). Overall, small intestinal organoids better replicate the structural and functional attributes of human small intestine tissues, offering an advanced system for analyzing drug effects in humans. As such, small intestinal organoids are expected to play a crucial role in upcoming drug screening initiatives.

### 5.4 Colorectal organoids

Mutations in the Wnt signaling pathway are observed in approximately 90% of colorectal cancers. Although numerous targeted therapies aimed at this pathway have been proposed, a subset of patients do not derive clinical benefit from these treatments. Kirsten rat sarcoma viral oncogene homolog (*KRAS*) mutations are prevalent in colorectal cancer. Verissimo et al. employed patient-derived organoids to evaluate therapeutic agents aimed at the EGFR-RAS-ERK signaling pathway. Their findings revealed that afatinib, an inhibitor of the epidermal growth factor receptor and human epidermal growth factor receptor (HER) 2, demonstrated efficacy against organoids with wild-type *KRAS* but was ineffective against those harboring *KRAS G12V* mutations. Additionally, the mitogen-activated protein kinase kinase (MEK) inhibitor selumetinib exhibited no therapeutic benefit as a standalone treatment. However, when combined with afatinib, MEK inhibitors were observed to increase the sensitivity of RAS-mutated tumors to HER2 inhibition (Verissimo et al., 2016). Moreover, simultaneous targeting of both the PI3K-AKT and EGFR-RAS-ERK pathways through the inhibition of phosphatidylinositol 3-kinase (PI3K) or serine/threonine protein kinase AKT, in conjunction with anti-EGFR therapy, did not improve treatment outcomes in *KRAS*-mutated tumors (Verissimo et al., 2016). As a result, the use of MEK inhibitors in conjunction with PI3K, AKT, or mammalian target of rapamycin (mTOR) inhibitors has not led to desirable outcomes in the treatment of *KRAS*-mutated colorectal cancer in clinical settings (Atanasova et al., 2023). The issue of drug resistance continues to be a major clinical obstacle, affecting roughly 40% of patients with *KRAS*-mutated colorectal cancer.

Knight et al. developed a colorectal cancer organoid harboring a *KRAS* mutation (Knight et al., 2021). They discovered that the combined inhibition of mitogen-activated protein kinase-interacting kinase (MNK) and mechanistic target of rapamycin complex 1 (mTORC1) enhanced the sensitivity of the organoids to rapamycin. The primary mechanism involves reducing the phosphorylation of eukaryotic translation initiation factor 4E and decreasing c-MYC expression, which may potentially inhibit tumor recurrence and metastasis in patients with *KRAS* mutations in clinical settings (Knight et al., 2021). Ringel et al. conducted CRISPR screening by integrating single guide RNAs from both wild-type and APC mutant human intestinal organoids (Ringel et al., 2020). By optimizing experimental conditions, they achieved a genome-wide CRISPR screen of organoids to identify tumor suppressor genes mediating TGF-β resistance, providing novel insights for drug development. Chimeric antigen receptor T-cell immunotherapy (CAR-T) has demonstrated promising therapeutic efficacy in leukemia. Consequently, Schnalzger et al. established PDOs of colon cancer to evaluate the cytotoxic effects of chimeric antigen receptors on solid tumors (Schnalzger et al., 2019). They found that engineered EGFRvIII-CAR NK-92 cells specifically targeted organoids transfected with the neoantigen EGFRvIII without exhibiting cytotoxicity towards normal organoids, suggesting that CAR-T technology may offer improved treatment options for colorectal cancer and other solid tumors. Ding et al. utilized droplet emulsification microfluidic technology to rapidly generate thousands of micro-organospheres (MOSs) from small colorectal cancer tissue samples. In this study, a total of eight MOSs derived from metastatic colorectal cancer patients were established. Four of these MOSs exhibited sensitivity to the drug, while the remaining four demonstrated resistance. Based on the drug sensitivity results, clinical treatment strategies were guided, and the drug responses of the sensitive and resistant MOSs were found to be consistent. Additionally, tumor stromal cells and immune cells, among other components of the tumor microenvironment, were detected within the MOSs. *In vitro* experiments showed that added T cells could infiltrate the MOSs and elicit a cytotoxic response to immunotherapy, thereby enhancing the killing effect on MOSs. This approach provides a platform for clinical trials to evaluate immuno-oncology therapies, including PD-1 blockade in patient tumors, bispecific antibodies, and T-cell therapies (Ding et al., 2022).

### 5.5 Liver organoids

Compared to traditional cell lines and xenograft models, organoid models exhibit superior performance in terms of construction success rate, culture duration, and preservation of disease characteristics. This makes them an invaluable tool for drug screening and adverse reaction research (Chen et al., 2024).

Li et al. utilized liver cancer organoids to screen 129 anticancer drugs, revealing that sorafenib, gemcitabine, and other antitumor agents demonstrated heterogeneous efficacy among liver cancer patients. They also identified pramikacin and idarubicin as potentially beneficial treatments for liver cancer (Li et al., 2019). Wang et al. employed organoids to investigate the mechanisms underlying sorafenib resistance (Wang et al., 2020). Kim et al. developed a MASH-related HCC mouse organoid model to evaluate drug responses, particularly Lenvatinib resistance (Kim et al., 2024b). Their findings indicated that while Multi-biotics (a soymilk fermented with lactic acid bacteria) did not directly inhibit tumor growth, it enhanced the efficacy of Lenvatinib, thereby indirectly suppressing tumor progression. Transcriptomic analysis revealed key pathways associated with *KRAS* signaling, inflammation, and epithelial-mesenchymal transition, identifying genes such as *Itga7*, *Col7a1*, and *Slpi* as potential targets to overcome Lenvatinib resistance. These insights provide valuable information on MASH-related HCC progression and drug resistance. Blukacz et al. demonstrated that inhibiting ABCB1, a drug efflux pump within the ABC transporter superfamily, increased adriamycin sensitivity in drug-resistant hepatocellular carcinoma organoids. They proposed combining adriamycin with ABCB1 inhibitors to enhance adriamycin efficacy and improve the response to transarterial chemoembolization (Blukacz et al., 2024). Collectively, these studies highlight the utility of organoids for *in vitro* drug sensitivity testing and the study of drug side effects.

### 5.6 Biliary organoids

Organoids are capable of accurately replicating the drug sensitivity and resistance characteristics seen in solid tissues. Additionally, they provide benefits like a brief preparation period and reliable passaging, which makes them highly useful for high-throughput drug screening (Yang and Yu, 2023).

Yuan et al. established a gallbladder cancer (GBC) organoid model through the co-culture of bile duct epithelial cells extracted from GBC with Matrigel (Yuan et al., 2022). Utilizing this model, they assessed the treatment potential of the dual PI3K/HDAC inhibitor CUDC-907 on GBC. Their results demonstrated that CUDC-907 effectively suppressed the growth of multiple GBC organoids and showed reduced toxicity to normal gallbladder organoids compared to other anticancer drugs in a double-controlled trial. These outcomes highlight the value of biliary organoids as tools for drug evaluation. Ren et al. managed to create cholangiocarcinoma organoids by isolating bile duct epithelial cells from cholangiocarcinoma tissues and culturing them together with Matrigel, achieving a high success rate (Ren et al., 2023). They further examined the therapeutic effects of seven frequently used chemotherapeutic agents—gemcitabine, cisplatin, capecitabine/5-fluorouracil, SN-38 (the active metabolite of irinotecan), oxaliplatin, mitomycin C, and paclitaxel—on these organoids. The results were then compared with follow-up data from cholangiocarcinoma patients and therapeutic outcomes from cholangiocarcinoma mouse models. The alignment across these three sets of findings further confirms the reliability and efficiency of biliary organoids as a platform for drug screening.

### 5.7 Pancreatic organoids

In recent years, the advent of targeted therapies, including immunotherapeutic agents, has significantly improved patient outcomes. However, a subset of patients remains unresponsive to current treatments due to tumor heterogeneity. This heterogeneity underscores the critical relationship between individual patient variability and drug efficacy, leading to diverse responses even among different cancer cells within the same tumor. Pancreatic cancer is often diagnosed at an advanced stage, with only 10%–15% of cases being amenable to surgical intervention. For patients with unresectable pancreatic cancer, organoid models offer a reliable platform for precision drug screening. High-throughput drug screening using organoids derived from pancreatic cancer tissues can facilitate the identification of effective therapeutic agents (Li et al., 2022).

**FIGURE 3:**
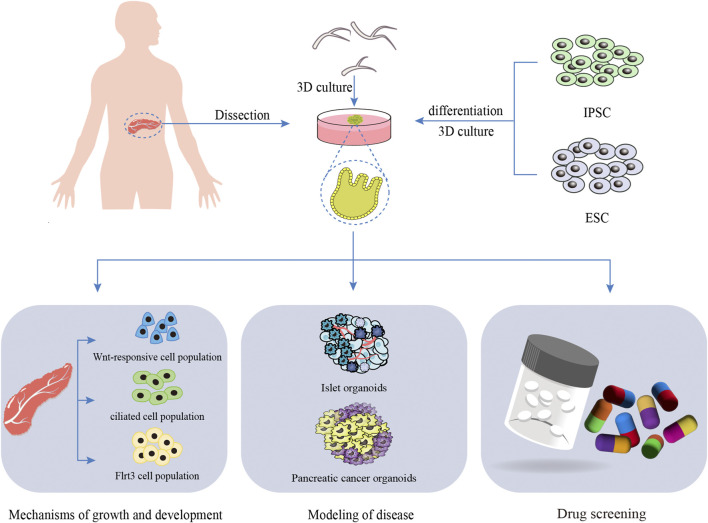
The application of pancreatic organoids. This diagram provides a comprehensive overview of the sources of seed cells for pancreatic organoids, including induced pluripotent stem cells (iPSC) and embryonic stem cells (ESC). It also details the applications of pancreatic organoids in elucidating the mechanisms of pancreatic growth and development, disease modeling, and drug screening.

Tiriac et al. utilized 156 patient-derived organoids to establish a platform for evaluating single-agent chemotherapy and targeted therapies, demonstrating that the therapeutic responses of pancreatic cancer organoids correlated with clinical outcomes in patients (Tiriac et al., 2018). Huang et al. developed a three-dimensional cell culture technique to expand and maintain primary pancreatic cancer organoids from patient tissues, enabling drug sensitivity testing (Huang et al., 2015). The researchers treated tumor organoids from five patients with gemcitabine and an epigenetic inhibitor, revealing differential drug sensitivity that correlated positively with resistance biomarkers. This study confirmed that pancreatic cancer organoids retained the sensitivity of patient tissues to novel agents *in vitro*. Hirt et al. selected 31 patient-derived pancreatic cancer organoids representing common genetic mutations and conducted high-throughput drug screening using an FDA-approved library of 1,172 compounds, including anti-tumor, cardiovascular, neurological, and anti-inflammatory drugs. Through automated drug administration, emetine and ouabain were identified as potential effective treatments, validated by *in vitro* and *in vivo* experiments. These compounds were found to induce tumor cell death by disrupting the hypoxic tolerance of pancreatic cancer organoids (Hirt et al., 2022). Watanabe et al. constructed a PDOX organoid model to screen for gemcitabine-sensitive and -resistant pancreatic cancer organoids. High-throughput screening of 375 kinase inhibitors was performed, and effective drugs were selected based on their efficacy and toxicity profiles (Watanabe et al., 2022). Zhou et al. demonstrated that the CLDN18.2/CD3 bispecific T cell engager (BiTE) effectively inhibited tumor growth in the early stages using a patient-derived organoid xenograft (PDOX) model. However, its efficacy markedly diminished in later stages. Notably, the combination of vilanterol and STING agonists synergistically enhanced BiTE efficacy by inhibiting CD64-positive CAFs and promoting the proliferation of stem-like CD8 T cells, thereby sustaining antitumor activity. Consequently, they proposed that the combination of vilanterol and STING agonists sensitizes PDAC to CLDN18.2-targeted BiTE therapy, enhancing its efficacy as a promising new strategy (Zhou et al., 2024b).In summary, these studies have validated the utility of organoid technology for investigating tumor heterogeneity, paving the way for establishing a living biobank of multiple patients’ tumor tissues to study individual pathogenic mechanisms, which is crucial for targeted research and personalized drug testing (Magré et al., 2023). The application of pancreatic organoids is shown in Figure 3.

As an emerging *in vitro* model, digestive system organoids have demonstrated significant potential in the field of drug screening. Oral organoids can serve as a disease model for oral cancer to investigate the anti-tumor effects of drugs. Through the establishment of gastric cancer organoids, metastatic colorectal cancer and pancreatic cancer cell line biobanks, large-scale drug screening has been performed, identifying compounds with notable sensitivity to organoid models. This provides valuable guidance for clinical drug selection and anti-cancer drug development. Drug-induced liver injury (DILI) represents one of the primary causes of clinical trial failure and high attrition rates in drug development. High-throughput generation of liver organoids can markedly accelerate the drug screening process and facilitate the discovery of novel therapeutics. The development of drug screening platforms based on microfluidic technology, in combination with pancreatic ductal adenocarcinoma (PDAC)-derived organoids, enables high-throughput drug screening and expedites drug discovery. However, organoid models still exhibit limitations in recapitulating the complexity of the *in vivo* microenvironment, such as the absence of key components like immune cells and the nervous system. Variability in organoids constructed across different laboratories may affect the reproducibility of drug screening results, highlighting the need for further research in this area.

## 6 Regenerative medicine

Currently, esophageal atresia, esophageal stenosis, esophageal cancer, and other conditions can be managed through esophagectomy. However, the use of distal gastrointestinal segments to reconstruct the resected esophagus often introduces significant inconvenience and new health challenges for patients. The relatively simple anatomical structure of the esophagus has facilitated the application of regenerative medicine in esophageal repair. Esophageal organoid units are an organoid system generated by seeding isolated esophageal cells in a Matrigel matrix gel and co-culturing them with neuromuscular cells (Spurrier et al., 2015). These organoids exhibit a gradient of epithelial differentiation from basal-like cells to mature squamous cells and can undergo spontaneous peristalsis. Spurrier et al. utilized esophageal organoid units in conjunction with tissue-engineered scaffolds, initially culturing esophageal progenitor cells *in vitro* before forming a tissue-engineered esophagus *in vivo* to achieve regenerative outcomes (Spurrier et al., 2015). This study demonstrated that esophageal organoids can serve as a viable cell source for esophageal regenerative medicine. Looking forward, esophageal organoids could potentially be integrated with 3D bioprinting technology to expand their applications in regenerative medicine.

Short bowel syndrome can result in the body’s inability to absorb sufficient nutrients, leading to intestinal failure. Intestinal transplantation, while a critical treatment option for such conditions, is associated with poor prognosis, including low long-term survival rates and the need for prolonged immunosuppression (Ueno et al., 2013). Therefore, it is imperative to develop more effective therapeutic approaches. Tissue engineering of the small intestine represents a promising alternative, with small intestinal organoid units providing essential cellular components for this process. In 2018, Hou et al. demonstrated that implanting mouse and human organoid units into mice could generate tissue-engineered intestines. After 3 months of *in vivo* development, these engineered tissues exhibited villus and crypt structures similar to those of adult small intestines, along with mature differentiation of small intestinal cells (Hou et al., 2018). A key advantage of these organoid units is their ability to maintain the expansion capacity of intestinal stem cells without exogenous growth factors, thereby minimizing the risk of carcinogenesis associated with added growth factors. In 2022, Lee et al. optimized the preservation conditions for small intestinal organoids by pretreating them with 5% dimethyl sulfoxide at 4°C for 30 min (Lee et al., 2022). Post-thawing, these organoids retained stable regenerative activity through continuous passage, enhancing storage technology for use in regenerative medicine. Although organoid units offer an alternative cell source for intestinal regenerative medicine, further experimental validation is required before transitioning to human studies.

**FIGURE 4:**
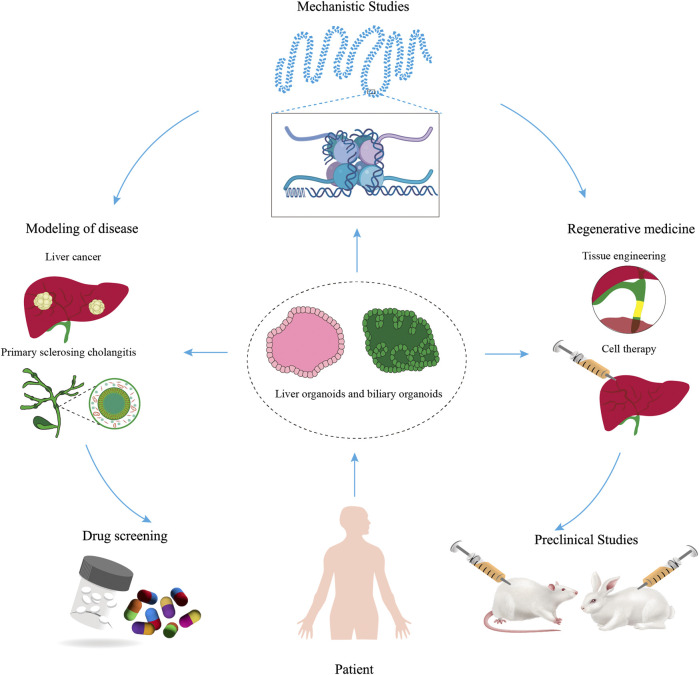
The applications of liver and biliary organoids. This figure offers a comprehensive overview of the diverse applications of liver and biliary organoids in disease modeling, mechanistic studies, drug screening, regenerative medicine, and preclinical investigations.

Despite orthotopic liver transplantation being an efficacious therapy for end-stage liver disease, its utility is markedly constrained by donor scarcity and the necessity for prolonged immunosuppression post-surgery. Liver organoids, as a scalable and functionally mature alternative, offer novel opportunities in regenerative medicine (Jalan-Sakrikar et al., 2023). Hepatic organoids can supply functional, genetically stable, proliferative cells capable of generating complex bioengineered tissues and integrating into the recipient’s vasculature (Kim et al., 2023). It is important to recognize that patients with end-stage liver disease often suffer from extensive damage to various cell types, including hepatocytes, rendering hepatocyte transplantation alone insufficient for complete liver function restoration. Consequently, multicellular organoid transplantation incorporating bile duct systems should be considered for repair. Liver organoids derived from ASCs or iPSCs of patients with end-stage liver disease may facilitate *in vivo* transplantation in the future, potentially treating liver failure, mitigating immune rejection, and enhancing graft survival. However, these organoids might exhibit diminished regenerative capacity due to underlying disease conditions. Optimizing the application of liver organoids in liver regenerative medicine remains a critical challenge. Currently, *in vivo* transplantation of organoids predominantly employs methods such as intrahepatic injection, splenic injection, renal subcapsular transplantation, or scaffold-based transplantation, all of which have limitations, including unpredictable cell distribution and low transplantation efficiency (Jalan-Sakrikar et al., 2023). Advances in tissue engineering and emerging biotechnologies, such as decellularized livers, 3D bioprinting, and organ-on-a-chip platforms, can be utilized to construct functional liver microtissues, providing cells with a microenvironment more closely resembling *in vivo* (Tabatabaei Rezaei et al., 2024; Huang et al., 2024b). Sampaziotis et al. successfully engineered organoids derived from human iPSCs-originated bile duct cells and transplanted them into the intrahepatic bile ducts of immunodeficient mice, leading to a significant improvement in the prognosis of mice with extrahepatic bile duct injuries (Sampaziotis et al., 2021). These findings provide a robust scientific basis for the potential of organoid transplantation. The synergistic advancement of liver organoids and biological tissue engineering has enhanced the feasibility of their application as grafts. Future research should focus on integrating liver organoids into the recipient liver at the vascular level to optimize graft functionality (Reza et al., 2021). The uses of liver and biliary organoids are depicted in Figure 4.

Digestive system organoids have a broad application prospect in regenerative medicine. Tissue repair and functional reconstruction are expected by transplanting organoids cultured *in vitro* into damaged tissues or organs. Radiotherapy often leads to salivary gland injury. The construction of salivary gland organoids by bio-printing technology is expected to provide a new strategy for the repair of damaged salivary glands. Small intestinal submucosa (SIS) has been widely used in tissue regeneration engineering, and its unique three-dimensional structure, biological function and low immunogenicity make it potential in repairing gastric mucosal injury (Barrile and Kasendra, 2025). In the aspect of hepatobiliary, hepatocyte transplantation alone is not enough to fully restore liver function, so multicellular organoid transplantation containing bile duct system is more effective. Despite the great potential of digestive system organoids in regenerative medicine, there are some challenges, such as the structural and functional complexity of organoids, vascularization issues, and stability in long-term culture. With the continuous development of technology, it is believed that these problems will be gradually solved, and digestive system organoids will play a greater role in the field of regenerative medicine.

## 7 Precision medicine

In the realm of precision medicine, high-throughput sequencing has emerged as a critical technique for detecting somatic mutations and driving the advancement of cancer-targeted therapies (Li et al., 2023a). The use of targeted drugs has not only improved overall survival rates among patients but also provided a new paradigm for personalized cancer treatment (Günther et al., 2022). Despite these advancements, the challenges in using genomic profiling to predict responses to targeted therapies, coupled with the limitations of preclinical models for validating drugs, have substantially hindered the progress of personalized medicine (Kim et al., 2024a; Tosca et al., 2023). There is now a pressing demand for *ex vivo* systems capable of reliably forecasting patient responses to therapeutic agents. Cancer stem cells, distinguished by their capacity for self-renewal and differentiation, present a potential solution through the creation of patient-derived organoids that accurately mimic tumor characteristics.Van de Wetering et al. conducted a proof-of-concept study to establish an organoid biobank from colorectal cancer patients, including both tumor and adjacent normal tissues. They performed high-throughput screening of 83 cancer drugs currently in clinical use or under investigation, including the anti-EGFR antibody cetuximab and first-line chemotherapeutic agents such as oxaliplatin and 5-fluorouracil. The study successfully evaluated drug-drug interactions within these organoids. It was found that tumors with *TP53* function loss exhibited resistance to murine double minute 2 inhibitors. Additionally, tumor organoids harboring activating *KRAS* mutations demonstrated resistance to anti-EGFR inhibitors (cetuximab and afatinib) (van de Wetering et al., 2015). Vlachogiannis and colleagues established a tumor organoid biobank using samples from patients with gastrointestinal metastatic tumors who had previously participated in phase I/II clinical trials (Vlachogiannis et al., 2018). Through comparing the reactions of organoids and orthotopic xenograft mouse models to the clinical trial responses of patients, they confirmed that organoids can faithfully mimic patient treatment results. This underscores the potential of organoids as a reliable system for drug testing. Precision oncology focuses on determining personalized anticancer treatments that are effective for individual patients (Hong et al., 2021).

Rectal cancer poses greater challenges compared to colon cancer due to its anatomical location within the pelvis and proximity to vital urogenital organs, complicating treatment approaches. Previous research has been limited by the absence of rectal cancer-specific cell lines, leading preclinical studies to rely on colon cancer cell lines. Ganesh et al. successfully established 65 rectal cancer organoids and demonstrated that the area under the dose-response curve for 5-fluorouracil and FOLFOX *in vitro* was negatively correlated with progression-free survival in corresponding clinical patients. This finding suggests that organoid drug sensitivity measurements can serve as a predictive tool to identify patients at risk of disease progression (Ganesh et al., 2019). Total mesorectal excision after neoadjuvant chemoradiotherapy continues to be the standard approach for treating locally advanced rectal cancer. Yao et al. established 80 organoids derived from patients with locally advanced rectal cancer to assess their response to 5-fluorouracil and irinotecan. Compared to clinical patient responses to neoadjuvant chemotherapy, this method achieved an accuracy of 84.43%, sensitivity of 78.01%, and specificity of 91.79%. These findings indicate that PDOs may offer novel therapeutic insights for locally advanced rectal cancer (Yao et al., 2020). However, Ooft et al.'s prospective clinical study on predicting chemotherapy response using metastatic colorectal cancer organoids yielded mixed results. While PDOs drug sensitivity tests could predict chemotherapy response in over 80% of patients treated with irinotecan, they failed to accurately predict outcomes for 5-fluorouracil plus oxaliplatin (Ooft et al., 2019). Consequently, PDOs can help prevent colorectal cancer patients from undergoing ineffective irinotecan chemotherapy. In conclusion, organoid technology is poised to play an increasingly significant role in the precision diagnosis and treatment of digestive diseases. Through the continuous optimization of organoid construction methods, coupled with the integration of multi-omics analysis and artificial intelligence technologies, it will be possible to provide patients with more personalized and precise treatment strategies.

## 8 Summary and prospect

Organoid technology offers a superior platform for elucidating the cellular and molecular biology of biliary tract tissues, as well as the pathogenesis and tumorigenesis mechanisms of digestive tract organs. This technology holds significant promise in various applications including disease modeling, drug screening, regenerative medicine, translational medicine, and research into physiological and pathological mechanisms. Organoids are initiated by stem cells that undergo division, differentiation, and self-assembly into multiple cell types. However, due to current technological limitations, organoids remain significantly smaller than their *in vivo* counterparts. Despite not being true human organs, organoids can closely mimic the structure and function of native tissues, rendering experimental data derived from organoids more reliable compared to traditional 2D cell lines and animal models. Moreover, patient-derived organoids hold potential for drug screening and personalized treatment strategies.

Presently, organoid technology in the digestive system has been successfully established, albeit with varying degrees of progress across different organs. Disease models for critical digestive organs such as the stomach, liver, and small intestine have covered numerous diseases, leading to the establishment of extensive organoid biobanks that facilitate comprehensive drug screening and other research endeavors. In contrast, esophageal organoid development lags behind other digestive organs, with current research primarily focused on esophageal cancer and limited exploration of other diseases. Additionally, oral organoid research remains in its infancy, largely confined to mouse-derived organoids, with human-derived organoid studies yet to be conducted.

Despite its numerous advantages, organoid technology still faces certain limitations. Firstly, the matrix gel utilized in organoid culture is primarily derived from the basement membrane matrix of Engelbreth-Holm-Swarm mouse sarcoma (Matrigel), which contains matrix proteins such as laminin, collagen IV, and nestin, along with various growth factors including TGF-β, epidermal growth factor, and insulin-like growth factor. Due to its tumor and murine origin, Matrigel cannot provide a standardized composition ratio and cannot establish an animal-free culture system, thereby limiting its clinical application. Secondly, organoids exhibit relatively low maturity, as they are deficient in vascular, lymphatic, and nervous system functions, allowing them to develop only fetal-like tissues instead of fully mature adult tissues. Lastly, differences in the culture conditions and techniques used for organoids might cause substantial alterations in cellular composition, thereby influencing organ differentiation and possibly compromising the consistency of experimental outcomes.

In the future, it is imperative to establish standardized culture protocols and quality control standards. Appropriate media and culture technologies should be selected based on specific requirements. Given the various limitations of Matrigel, alternative matrices such as animal- and plant-derived gels, as well as synthetic macromolecular polymer gels, can be utilized for gastrointestinal organoid cultures (Zeiringer et al., 2023; Hunt et al., 2021; Curvello et al., 2020). As a critical component in 3D organoid culture, matrix materials require further exploration to meet the diverse demands within regenerative medicine. Three-dimensional vascularized organoids generated through organ-on-a-chip technology facilitate flux generation, vascularization, organoid interaction, and tissue microenvironment control, thereby guiding stem cell growth, differentiation, and organoid morphogenesis while overcoming existing research limitations (Monteduro et al., 2023). To ensure that organoids receive adequate oxygen and nutrients while effectively discharging metabolic waste, vascularization must be introduced. Microvascular networks can be constructed using 3D printing technology or biomaterial scaffolds (Su et al., 2022), and vascular endothelial cells can be differentiated from stem cells to promote angiogenesis (Majid et al., 2024). In addition to vascularization, incorporating appropriate neural connections is crucial for enhancing organoid functionality. This can be achieved by employing gene editing tools like CRISPR-Cas9 to program cells to express specific nerve growth factors or signaling molecules that promote the extension of nerve fibers to target areas and establish functional connections, thus forming natural neural networks (Testa et al., 2022). To further enhance the simulation effect of organoids, interactions between multiple types of organoids must also be considered. For instance, liver-kidney co-culture systems can provide deeper insights into drug metabolism processes and their effects on the human body (Huang et al., 2024a).

The three-dimensional architecture of organoids renders them a superior platform for disease modeling and drug screening compared to traditional two-dimensional cell lines. The 3D structure of organoid-based disease models offers significant advantages, enabling more accurate representation of *in vivo* conditions. Recent studies have successfully utilized organoid technology to facilitate cross-referencing and comparative analyses across various models. The feasibility of employing organoids for drug screening has been demonstrated, with their structural and functional resemblance to human tissues positioning them as promising platforms for pharmaceutical research (Piraino et al., 2024). PDOs have promising prospects in the field of personalized medicine, and organoid technology could be crucial in driving the development of precision medicine. Furthermore, owing to their regenerative and proliferative capabilities, organoids exhibit substantial potential in the field of regenerative medicine (Wu et al., 2023b).

With the deepening application of artificial intelligence (AI) in medicine and biotechnology, particularly in gastrointestinal organoid research, AI technology has demonstrated significant potential. Firstly, the study of gastrointestinal organoids has generated extensive bioinformatic data encompassing genomics, proteomics, metabolomics, and clinical information. AI can process and analyze this vast dataset, uncover hidden correlations, and assist scientists in identifying novel biomarkers and disease mechanisms. Secondly, AI can predict cell growth, differentiation, and behavior under various conditions, aiding researchers in optimizing culture conditions for GI organoids and enhancing their stability and functionality. Thirdly, by analyzing organoid response patterns and predicting drug effects, AI can expedite the drug screening process and reduce the time and cost associated with drug development. Finally, AI can assess the biological characteristics of gastrointestinal organoids, predict individual risk for specific gastrointestinal diseases, and provide a foundation for early intervention. Nevertheless, the application of AI in organoid studies is still in its early stages. Developing trustworthy databases and enhancing AI models remain essential objectives. In the future, there is hope that an AI-powered organoid automation platform, encompassing automated cultivation, surveillance, and evaluation, will greatly boost experimental effectiveness and accuracy. The capabilities of artificial intelligence are anticipated to propel the advancement of gastrointestinal organoids to new heights. Overall, ongoing advancements in organoid technology will be crucial for achieving more sophisticated organ functionalities, thus strengthening their utility in disease simulation, pharmaceutical testing, and regenerative therapies.

## Glossary

2D: Two-dimensional
3D: Three-dimensional
4D: Four-dimensional
ASCs: Adult stem cells
ABCB1: A drug efflux pump within the ABC transporter superfamily
BA: Biliary atresia
BACOs: Bile duct atresia cystic organoids
BE: Barrett’s esophagus
BiTE: Bispecific T cell engager
CAFs: Cancer-associated fibroblasts
CAR-T: Chimeric antigen receptor T-cell immunotherapy
CEACAM1: Carcinoembryonic antigen-related cell adhesion molecule 1
CFTR: Cystic fibrosis transmembrane conductance regulator
CRISPR-Cas9: Clustered regularly interspaced short palindromic repeats
EAC: Esophageal adenocarcinoma
ECM: Extracellular matrix
EGFR: Epidermal growth factor receptor
ESCC: Esophageal squamous cell carcinoma
ESCs: Embryonic stem cells
FGF: Fibroblast growth factor
FPCO: Fused Pancreatic Cancer Organoid
Fzd: Wnt Frizzled receptors
GBC: Gallbladder cancer
GEP-NEN: Gastrointestinal pancreatic neuroendocrine neoplasms
hAEC: Human amniotic epithelial cells
HBV: Hepatitis B virus
HCC: Hepatocellular carcinoma
HDAC: Histone deacetylase
HER: Human epidermal growth factor receptor
hfPOs: Human fetal pancreatic organoid
hiPSCs: Human induced pluripotent stem cells
HNSCC: Head and neck squamous cell carcinoma
Hv1Lt1: Humanized anti-CKAP4 antibody
KRAS: Kirsten rat sarcoma viral oncogene homolog
ISCs: Epithelial stem cells
MAPK: Mitogen-activated protein kinase
MCT1: Monocarboxylate transporter 1
MEK: Mitogen-activated protein kinase kinase
MNK: Mitogen-activated protein kinase-interacting kinase
MOSs: Micro-organospheres
mTOR: Mammalian target of rapamycin
mTORC1: Mechanistic target of rapamycin complex 1
NASH: Non-alcoholic steatohepatitis
NAFLD: Non-alcoholic fatty liver disease
iPSCs: Induced pluripotent stem cells
OSCC: Oral squamous cell carcinoma
PDAC: Pancreatic ductal adenocarcinoma
PDOs: Patient-derived organoids
PI3K: Phosphatidylinositol 3-kinase
PSCs: Pluripotent stem cells
STAT3: Signal transducer and activator of transcription 3
TAMs: Tumor-associated macrophages
TGF: Transforming growth factor
Yap1: Yes-associated protein 1

## References

[B1] Abbasalizadeh S. Babaee S. Kowsari-Esfahan R. Mazidi Z. Shi Y. Wainer J. (2023). Continuous production of highly functional vascularized hepatobiliary organoids from human pluripotent stem cells using a scalable microfluidic platform. Adv. Funct. Mater. 33, 2210233. 10.1002/adfm.202210233 PMC12382460 40881154

[B2] Adpaikar A. A. Zhang S. Kim H. Y. Kim K. W. Moon S. J. Lee J. M. (2022). Fine-tuning of epithelial taste bud organoid to promote functional recapitulation of taste reactivity. Cell Mol. Life Sci. 79, 211. 10.1007/s00018-022-04242-0 35344108 PMC8958342

[B3] Afonso M. B. Marques V. VAN Mil S. W. C. Rodrigues C. M. P. (2024). Human liver organoids: from generation to applications. Hepatology 79, 1432–1451. 10.1097/HEP.0000000000000343 36815360 PMC11095893

[B4] AL-Qadami G. Raposo A. Chien C.-C. Ma C. Priebe I. Hor M. (2025). Intestinal organoid coculture systems: current approaches, challenges, and future directions. Am. J. Physiology-Gastrointestinal Liver Physiology 328, G252–G276. 10.1152/ajpgi.00203.2024 39716040

[B5] Amarachintha S. P. Mourya R. Ayabe H. Yang L. Luo Z. Li X. (2022). Biliary organoids uncover delayed epithelial development and barrier function in biliary atresia. Hepatology 75, 89–103. 10.1002/hep.32107 34392560 PMC9983428

[B6] Andersson-Rolf A. Groot K. Korving J. Begthel H. Hanegraaf M. A. J. Vaninsberghe M. (2024). Long-term *in vitro* expansion of a human fetal pancreas stem cell that generates all three pancreatic cell lineages. Cell 187, 7394–7413.e22. 10.1016/j.cell.2024.10.044 39626658

[B7] Assad H. Assad A. Kumar A. (2023). Recent developments in 3D bio-printing and its biomedical applications. Pharmaceutics 15, 255. 10.3390/pharmaceutics15010255 36678884 PMC9861443

[B8] Atanasova V. S. Riedl A. Strobl M. Flandorfer J. Unterleuthner D. Weindorfer C. (2023). Selective eradication of colon cancer cells harboring PI3K and/or MAPK pathway mutations in 3D culture by combined PI3K/AKT/mTOR pathway and MEK inhibition. Int. J. Mol. Sci. 24, 1668. 10.3390/ijms24021668 36675180 PMC9863259

[B9] Bailey P. Chang D. K. Nones K. Johns A. L. Patch A.-M. Gingras M.-C. (2016). Genomic analyses identify molecular subtypes of pancreatic cancer. Nature 531, 47–52. 10.1038/nature16965 26909576

[B10] Baptista L. S. Porrini C. G S. K. Kelly D. J. Perrault C. M. (2024). Corrigendum: 3D organ-on-a-chip: the convergence of microphysiological systems and organoids. Front. Cell Dev. Biol. 12, 1365671. 10.3389/fcell.2024.1365671 38344748 PMC10853685

[B11] Barrile R. Kasendra M. (2025). Shaping intestinal organoids: engineering crypt curvature to guide stem cell niches. Cell Stem Cell 32, 678–680. 10.1016/j.stem.2025.03.015 40315831

[B12] Barron M. R. Cieza R. J. Hill D. R. Huang S. Yadagiri V. K. Spence J. R. (2020). The lumen of human intestinal organoids poses greater stress to bacteria compared to the germ-free mouse intestine: *Escherichia coli* deficient in RpoS as a colonization probe. mSphere 5. 10.1128/mSphere.00777-20 PMC7657587 33177212

[B13] Basak O. Beumer J. Wiebrands K. Seno H. VAN Oudenaarden A. Clevers H. (2017). Induced quiescence of Lgr5+ stem cells in intestinal organoids enables differentiation of hormone-producing enteroendocrine cells. Cell Stem Cell 20, 177–190. 10.1016/j.stem.2016.11.001 27939219

[B14] Bassi G. Grimaudo M. A. Panseri S. Montesi M. (2021). Advanced multi-dimensional cellular models as emerging reality to reproduce *in vitro* the human body complexity. Int. J. Mol. Sci. 22, 1195. 10.3390/ijms22031195 33530487 PMC7865724

[B15] Bein A. Shin W. Jalili-Firoozinezhad S. Park M. H. Sontheimer-Phelps A. Tovaglieri A. (2018). Microfluidic organ-on-a-chip models of human intestine. Cell Mol. Gastroenterol. Hepatol. 5, 659–668. 10.1016/j.jcmgh.2017.12.010 29713674 PMC5924739

[B16] Belair D. G. Wolf C. J. Moorefield S. D. Wood C. Becker C. Abbott B. D. (2018). A three-dimensional organoid culture model to assess the influence of chemicals on morphogenetic fusion. Toxicol. Sci. 166, 394–408. 10.1093/toxsci/kfy207 30496568

[B17] Below C. R. Kelly J. Brown A. Humphries J. D. Hutton C. Xu J. (2022). A microenvironment-inspired synthetic three-dimensional model for pancreatic ductal adenocarcinoma organoids. Nat. Mater. 21, 110–119. 10.1038/s41563-021-01085-1 34518665 PMC7612137

[B18] Blukacz L. Nuciforo S. Fucile G. Trulsson F. Duthaler U. Wieland S. (2024). Inhibition of the transmembrane transporter ABCB1 overcomes resistance to doxorubicin in patient-derived organoid models of HCC. Hepatol. Commun. 8, e0437. 10.1097/HC9.0000000000000437 38696353 PMC11068137

[B19] Bradney M. J. Venis S. M. Yang Y. Konieczny S. F. Han B. (2020). A biomimetic tumor model of heterogeneous invasion in pancreatic ductal adenocarcinoma. Small 16, e1905500. 10.1002/smll.201905500 31997571 PMC7069790

[B20] Cabeza-Segura M. Garcia-Micò B. LA Noce M. Nicoletti G. F. Conti V. Filippelli A. (2023). How organoids can improve personalized treatment in patients with gastro-esophageal tumors. Curr. Opin. Pharmacol. 69, 102348. 10.1016/j.coph.2023.102348 36842387

[B21] Chadwick M. Yang C. Liu L. Gamboa C. M. Jara K. Lee H. (2020). Rapid processing and drug evaluation in glioblastoma patient-derived organoid models with 4D bioprinted arrays. iScience 23, 101365. 10.1016/j.isci.2020.101365 32731171 PMC7393526

[B22] Chan M. C. Cheung S. K. C. Mohammad K. N. Chan J. C. M. Estes M. K. Chan P. K. S. (2019). Use of human intestinal enteroids to detect human norovirus infectivity. Emerg. Infect. Dis. 25, 1730–1735. 10.3201/eid2509.190205 31441758 PMC6711227

[B23] Chen S. Li P. Wang Y. Yin Y. DE Ruiter P. E. Verstegen M. M. A. (2020). Rotavirus infection and cytopathogenesis in human biliary organoids potentially recapitulate biliary atresia development. mBio 11. 10.1128/mBio.01968-20 PMC7448284 32843549

[B24] Chen Z. Long L. Wang J. Li W. Wang A. Kankala R. K. (2024). Constructing tumor organoid-like tissue for reliable drug screening using liver-decellularized extracellular matrix scaffolds. ACS Omega 9, 5888–5898. 10.1021/acsomega.3c09265 38343980 PMC10851408

[B25] Cherne M. D. Sidar B. Sebrell T. A. Sanchez H. S. Heaton K. Kassama F. J. (2021). A synthetic hydrogel, VitroGel(®) ORGANOID-3, improves immune cell-epithelial interactions in a tissue chip Co-culture model of human gastric organoids and dendritic cells. Front. Pharmacol. 12, 707891. 10.3389/fphar.2021.707891 34552484 PMC8450338

[B26] Cherubini A. Rusconi F. Piras R. WäCHTERSHäUSER K. N. Dossena M. Barilani M. (2024). Exploring human pancreatic organoid modelling through single-cell RNA sequencing analysis. Commun. Biol. 7, 1527. 10.1038/s42003-024-07193-3 39558019 PMC11574267

[B27] Chi K. Y. Kim G. Kim H. Kim H. Jo S. Lee J. (2025). Optimization of culture conditions to generate vascularized multi-lineage liver organoids with structural complexity and functionality. Biomaterials 314, 122898. 10.1016/j.biomaterials.2024.122898 39447308

[B28] Christine Verawaty Sibuea J. P. R. A. C. O. M. J. I. R. S. E. L. R. W. N. W. M. N. F. M. Pawitan J. Antarianto R. Jasirwan C. O. Sianipar I. R. Luviah E. (2020). 3D Co-culture of hepatocyte, a hepatic stellate cell line, and stem cells for developing a bioartificial liver prototype. Int. J. Technol. 11, 951–319. 10.14716/ijtech.v11i5.4317

[B29] Curvello R. Kerr G. Micati D. J. Chan W. H. Raghuwanshi V. S. Rosenbluh J. (2020). Engineered plant-based nanocellulose hydrogel for small intestinal organoid growth. Adv. Sci. (Weinh) 8, 2002135. 10.1002/advs.202002135 33437574 PMC7788499

[B30] DE Crignis E. Hossain T. Romal S. Carofiglio F. Moulos P. Khalid M. M. (2021). Application of human liver organoids as a patient-derived primary model for HBV infection and related hepatocellular carcinoma. Elife 10, e60747. 10.7554/eLife.60747 34328417 PMC8384419

[B31] Dekkers J. F. VAN DER Ent C. K. Beekman J. M. (2013). Novel opportunities for CFTR-targeting drug development using organoids. Rare Dis. 1, e27112. 10.4161/rdis.27112 25003014 PMC3915567

[B32] Deng S. Li C. Cao J. Cui Z. DU J. Fu Z. (2023). Organ-on-a-chip meets artificial intelligence in drug evaluation. Theranostics 13, 4526–4558. 10.7150/thno.87266 37649608 PMC10465229

[B33] Ding S. Hsu C. Wang Z. Natesh N. R. Millen R. Negrete M. (2022). Patient-derived micro-organospheres enable clinical precision oncology. Cell Stem Cell 29, 905–917.e6. 10.1016/j.stem.2022.04.006 35508177 PMC9177814

[B34] Driehuis E. Kolders S. Spelier S. Lohmussaar K. Willems S. M. Devriese L. A. (2020a). Correction: oral mucosal organoids as a potential platform for personalized cancer therapy. Cancer Discov. 10, 476. 10.1158/2159-8290.CD-20-0129 31053628

[B35] Driehuis E. Oosterom N. Heil S. G. Muller I. B. Lin M. Kolders S. (2020b). Patient-derived oral mucosa organoids as an *in vitro* model for methotrexate induced toxicity in pediatric acute lymphoblastic leukemia. PLoS One 15, e0231588. 10.1371/journal.pone.0231588 32421698 PMC7233536

[B36] DU Y. DE Jong I. E. M. Gupta K. Waisbourd-Zinman O. Har-Zahav A. Soroka C. J. (2023). Human vascularized bile duct-on-a chip: a multi-cellular micro-physiological system for studying cholestatic liver disease. Biofabrication 16, 015004. 10.1088/1758-5090/ad0261 PMC10587873 37820623

[B37] DU Y. Khandekar G. Llewellyn J. Polacheck W. Chen C. S. Wells R. G. (2020). A bile duct-on-a-chip with organ-level functions. Hepatology 71, 1350–1363. 10.1002/hep.30918 31465556 PMC7048662

[B38] Fang G. Lu H. AL-Nakashli R. Chapman R. Zhang Y. Ju L. A. (2021). Enabling peristalsis of human colon tumor organoids on microfluidic chips. Biofabrication 14, 015006. 10.1088/1758-5090/ac2ef9 34638112

[B39] FernáNDEZ Á. Casamitjana J. HolguíN-Horcajo A. Coolens K. Mularoni L. Guo L. (2024). A single-cell atlas of the murine pancreatic ductal tree identifies novel cell populations with potential implications in pancreas regeneration and exocrine pathogenesis. Gastroenterology 167, 944–960.e15. 10.1053/j.gastro.2024.06.008 38908487

[B40] Finkbeiner S. R. Zeng X. L. Utama B. Atmar R. L. Shroyer N. F. Estes M. K. (2012). Stem cell-derived human intestinal organoids as an infection model for rotaviruses. mBio 3, e00159–e00112. 10.1128/mBio.00159-12 22761392 PMC3398537

[B41] Flood P. Hanrahan N. Nally K. Melgar S. (2024). Human intestinal organoids: modeling gastrointestinal physiology and immunopathology - current applications and limitations. Eur. J. Immunol. 54, e2250248. 10.1002/eji.202250248 37957831

[B42] Flynn T. G. Olortegui M. P. Kosek M. N. (2024). Viral gastroenteritis. Lancet 403, 862–876. 10.1016/S0140-6736(23)02037-8 38340741

[B43] Friedman S. L. Pinzani M. (2022). Hepatic fibrosis 2022: unmet needs and a blueprint for the future. Hepatology 75, 473–488. 10.1002/hep.32285 34923653 PMC12179971

[B44] Fujii M. Matano M. Toshimitsu K. Takano A. Mikami Y. Nishikori S. (2018). Human intestinal organoids maintain self-renewal capacity and cellular diversity in niche-inspired culture condition. Cell Stem Cell 23, 787–793. 10.1016/j.stem.2018.11.016 30526881

[B45] Fujii M. Sato T. (2021). Somatic cell-derived organoids as prototypes of human epithelial tissues and diseases. Nat. Mater 20, 156–169. 10.1038/s41563-020-0754-0 32807924

[B46] Ganesh K. Wu C. O'Rourke K. P. Szeglin B. C. Zheng Y. Sauvé C. G. (2019). A rectal cancer organoid platform to study individual responses to chemoradiation. Nat. Med. 25, 1607–1614. 10.1038/s41591-019-0584-2 31591597 PMC7385919

[B47] Ghorbaninejad M. Asadzadeh-Aghdaei H. Baharvand H. Meyfour A. (2023). Intestinal organoids: a versatile platform for modeling gastrointestinal diseases and monitoring epigenetic alterations. Life Sci. 319, 121506. 10.1016/j.lfs.2023.121506 36858311

[B48] GüNTHER C. Winner B. Neurath M. F. Stappenbeck T. S. (2022). Organoids in gastrointestinal diseases: from experimental models to clinical translation. Gut 71, 1892–1908. 10.1136/gutjnl-2021-326560 35636923 PMC9380493

[B49] Guo H. Urban S. Wang W. (2023). *In vitro* cell culture models to study hepatitis B and D virus infection. Front. Microbiol. 14, 1169770. 10.3389/fmicb.2023.1169770 37089540 PMC10113554

[B50] Habanjar O. Diab-Assaf M. Caldefie-Chezet F. Delort L. (2021). 3D cell culture systems: tumor application, advantages, and disadvantages. Int. J. Mol. Sci. 22, 12200. 10.3390/ijms222212200 34830082 PMC8618305

[B51] Hamilton M. Jean D. Boudreau F. Giroux V. (2022). A21 Overexpression of ASCL2 alters differentiation in esophageal organoids. J. Can. Assoc. Gastroenterology 5, 24–25. 10.1093/jcag/gwab049.020

[B52] Haque M. R. Rempert T. H. AL-Hilal T. A. Wang C. Bhushan A. Bishehsari F. (2021). Organ-chip models: opportunities for precision medicine in pancreatic cancer. Cancers (Basel) 13, 4487. 10.3390/cancers13174487 34503294 PMC8430573

[B53] He J. Cui H. Y. Shi X. H. Jin Q. Q. Han X. M. Han T. T. (2022). Functional hepatobiliary organoids recapitulate liver development and reveal essential drivers of hepatobiliary cell fate determination. LIFE Med. 1, 345–358. 10.1093/lifemedi/lnac055 39872746 PMC11749142

[B54] Hemeryck L. Hermans F. Chappell J. Kobayashi H. Lambrechts D. Lambrichts I. (2022). Organoids from human tooth showing epithelial stemness phenotype and differentiation potential. Cell Mol. Life Sci. 79, 153. 10.1007/s00018-022-04183-8 35217915 PMC8881251

[B55] Hermans F. Hemeryck L. Bueds C. Torres Pereiro M. Hasevoets S. Kobayashi H. (2023). Organoids from mouse molar and incisor as new tools to study tooth-specific biology and development. Stem Cell Rep. 18, 1166–1181. 10.1016/j.stemcr.2023.03.011 PMC10202652 37084723

[B56] Hirokawa Y. Clarke J. Palmieri M. Tan T. Mouradov D. Li S. (2021). Low-viscosity matrix suspension culture enables scalable analysis of patient-derived organoids and tumoroids from the large intestine. Commun. Biol. 4, 1067. 10.1038/s42003-021-02607-y 34518628 PMC8438070

[B57] Hirt C. K. Booij T. H. Grob L. Simmler P. Toussaint N. C. Keller D. (2022). Drug screening and genome editing in human pancreatic cancer organoids identifies drug-gene interactions and candidates for off-label treatment. Cell Genom 2, 100095. 10.1016/j.xgen.2022.100095 35187519 PMC7612395

[B58] Hockney S. Parker J. Turner J. E. Todd X. Todryk S. Gieling R. G. (2023). Next generation organoid engineering to replace animals in cancer drug testing. Biochem. Pharmacol. 213, 115586. 10.1016/j.bcp.2023.115586 37164297

[B59] Hong H. K. Yun N. H. Jeong Y. L. Park J. Doh J. Lee W. Y. (2021). Establishment of patient-derived organotypic tumor spheroid models for tumor microenvironment modeling. Cancer Med. 10, 5589–5598. 10.1002/cam4.4114 34240815 PMC8366099

[B60] Hou X. Chang D. F. Trecartin A. Barthel E. R. Schlieve C. R. Frey M. R. (2018). Short-term and long-term human or mouse organoid units generate tissue-engineered small intestine without added signalling molecules. Exp. Physiol. 103, 1633–1644. 10.1113/EP086990 30232817

[B61] Huang L. Holtzinger A. Jagan I. Begora M. Lohse I. Ngai N. (2015). Ductal pancreatic cancer modeling and drug screening using human pluripotent stem cell– and patient-derived tumor organoids. Nat. Med. 21, 1364–1371. 10.1038/nm.3973 26501191 PMC4753163

[B62] Huang Q. Yang T. Song Y. Sun W. Xu J. Cheng Y. (2024a). A three-dimensional (3D) liver-kidney on a chip with a biomimicking circulating system for drug safety evaluation. Lab. Chip 24, 1715–1726. 10.1039/d3lc00980g 38328873

[B63] Huang Y. Liu T. Huang Q. Wang Y. (2024b). From organ-on-a-chip to human-on-a-chip: a review of research progress and latest applications. ACS Sens. 9, 3466–3488. 10.1021/acssensors.4c00004 38991227

[B64] Hunt D. R. Klett K. C. Mascharak S. Wang H. Gong D. Lou J. (2021). Engineered matrices enable the culture of human patient-derived intestinal organoids. Adv. Sci. (Weinh) 8, 2004705. 10.1002/advs.202004705 34026461 PMC8132048

[B65] Huycke T. R. HäKKINEN T. J. Miyazaki H. Srivastava V. Barruet E. Mcginnis C. S. (2024). Patterning and folding of intestinal villi by active mesenchymal dewetting. Cell 187, 3072–3089.e20. 10.1016/j.cell.2024.04.039 38781967 PMC11166531

[B66] Idowu S. Bertrand P. P. Walduck A. K. (2022). Gastric organoids: advancing the study of *H. pylori* pathogenesis and inflammation. Helicobacter 27, e12891. 10.1111/hel.12891 35384141 PMC9287064

[B67] Jalan-Sakrikar N. Brevini T. Huebert R. C. Sampaziotis F. (2023). Organoids and regenerative hepatology. Hepatology 77, 305–322. 10.1002/hep.32583 35596930 PMC9676408

[B68] Jalan-Sakrikar N. DE Assuncao T. M. Navarro-Corcuera A. Hamdan F. H. Huebert R. C. (2022). Induced pluripotent stem cells from subjects with primary sclerosing cholangitis develop a senescence phenotype following biliary differentiation. Hepatol. Commun. 6, 345–360. 10.1002/hep4.1809 34519176 PMC8793999

[B69] Jangra A. Kothari A. Sarma P. Medhi B. Omar B. J. Kaushal K. (2022). Recent advancements in antifibrotic therapies for regression of liver fibrosis. Cells 11, 1500. 10.3390/cells11091500 35563807 PMC9104939

[B70] Jing S. Lian L. Hou Y. Li Z. Zheng Z. Li G. (2023). Advances in volumetric bioprinting. Biofabrication 16, 012004. 10.1088/1758-5090/ad0978 37922535

[B71] Ji S. Feng L. Fu Z. Wu G. Wu Y. Lin Y. (2023). Pharmaco-proteogenomic characterization of liver cancer organoids for precision oncology. Sci. Transl. Med. 15, eadg3358. 10.1126/scitranslmed.adg3358 37494474 PMC10949980

[B72] Kalogeropoulou M. DíAZ-Payno P. J. Mirzaali M. J. VAN Osch G. Fratila-Apachitei L. E. Zadpoor A. A. (2024). 4D printed shape-shifting biomaterials for tissue engineering and regenerative medicine applications. Biofabrication 16, 022002. 10.1088/1758-5090/ad1e6f 38224616

[B73] Kantaros A. (2022). 3D printing in regenerative medicine: technologies and resources utilized. Int. J. Mol. Sci. 23, 14621. 10.3390/ijms232314621 36498949 PMC9738732

[B74] Karakasheva T. A. Kijima T. Shimonosono M. Maekawa H. Sahu V. Gabre J. T. (2020). Generation and characterization of patient-derived head and neck, oral, and esophageal cancer organoids. Curr. Protoc. Stem Cell Biol. 53, e109. 10.1002/cpsc.109 32294323 PMC7350550

[B75] Kasagi Y. Chandramouleeswaran P. M. Whelan K. A. Tanaka K. Giroux V. Sharma M. (2018). The esophageal organoid system reveals functional interplay between Notch and cytokines in reactive epithelial changes. Cell Mol. Gastroenterol. Hepatol. 5, 333–352. 10.1016/j.jcmgh.2017.12.013 29552622 PMC5852293

[B76] Kawasaki K. Toshimitsu K. Matano M. Fujita M. Fujii M. Togasaki K. (2020). An organoid biobank of neuroendocrine neoplasms enables genotype-phenotype mapping. Cell 183, 1420–1435. 10.1016/j.cell.2020.10.023 33159857

[B77] Khedr M. A. Mohamed Z. EL-Derby A. M. Soliman M. M. Edris A. A. F. Badr E. (2024). Development of hepatocellular carcinoma organoid model recapitulating HIF-1A metabolic signature. Clin. Exp. Med. 25, 9. 10.1007/s10238-024-01521-x 39567394 PMC11579110

[B78] Kijima T. Nakagawa H. Shimonosono M. Chandramouleeswaran P. M. Hara T. Natsugoe S. (2019). Three-dimensional organoids reveal therapy resistance of esophageal and oropharyngeal squamous cell carcinoma cells. Cell Mol. Gastroenterol. Hepatol. 7, 73–91. 10.1016/j.jcmgh.2018.09.003 30510992 PMC6260338

[B79] Kim H. J. Kim G. Chi K. Y. Kim H. Jang Y. J. Jo S. (2023). Generation of multilineage liver organoids with luminal vasculature and bile ducts from human pluripotent stem cells via modulation of Notch signaling. Stem Cell Res. Ther. 14, 19. 10.1186/s13287-023-03235-5 36737811 PMC9898924

[B80] Kim J. Koo B. K. Knoblich J. A. (2020). Human organoids: model systems for human biology and medicine. Nat. Rev. Mol. Cell Biol. 21, 571–584. 10.1038/s41580-020-0259-3 32636524 PMC7339799

[B81] Kim J. Park S. H. Lee H. Kim Y. Cho K. H. (2024a). PANCDR: precise medicine prediction using an adversarial network for cancer drug response. Brief. Bioinform 25, bbae406. 10.1093/bib/bbae406 38487849 PMC10940842

[B82] Kim S. Jeong N. Park J. Noh H. Lee J. O. Yu S. J. (2024b). Establishment and characterization of mouse metabolic dysfunction-associated steatohepatitis-related hepatocellular carcinoma organoids. Sci. Rep. 14, 27460. 10.1038/s41598-024-78963-6 39523389 PMC11551198

[B83] Kimura M. Iguchi T. Iwasawa K. Dunn A. Thompson W. L. Yoneyama Y. (2022). En masse organoid phenotyping informs metabolic-associated genetic susceptibility to NASH. Cell 185, 4216–4232.e16. 10.1016/j.cell.2022.09.031 36240780 PMC9617783

[B84] Knight J. R. P. Alexandrou C. Skalka G. L. Vlahov N. Pennel K. Officer L. (2021). MNK inhibition sensitizes KRAS-mutant colorectal cancer to mTORC1 inhibition by reducing eIF4E phosphorylation and c-MYC expression. Cancer Discov. 11, 1228–1247. 10.1158/2159-8290.CD-20-0652 33328217 PMC7611341

[B85] Kozlowski M. T. Crook C. J. Ku H. T. (2021). Towards organoid culture without Matrigel. Commun. Biol. 4, 1387. 10.1038/s42003-021-02910-8 34893703 PMC8664924

[B86] Kunze B. Wein F. Fang H. Y. Anand A. Baumeister T. Strangmann J. (2020). Notch signaling mediates differentiation in barrett's esophagus and promotes progression to adenocarcinoma. Gastroenterology 159, 575–590. 10.1053/j.gastro.2020.04.033 32325086 PMC7484392

[B87] Kwon O. Lee H. Jung J. Son Y. S. Jeon S. Yoo W. D. (2024). Chemically-defined and scalable culture system for intestinal stem cells derived from human intestinal organoids. Nat. Commun. 15, 799. 10.1038/s41467-024-45103-7 38280855 PMC10821882

[B88] Lazarus J. V. Mark H. E. Allen A. M. Arab J. P. Carrieri P. Noureddin M. (2023). A global research priority agenda to advance public health responses to fatty liver disease. J. Hepatol. 79, 618–634. 10.1016/j.jhep.2023.04.035 37353401

[B89] Lebreton F. Bellofatto K. Wassmer C. H. Perez L. Lavallard V. Parnaud G. (2020). Shielding islets with human amniotic epithelial cells enhances islet engraftment and revascularization in a murine diabetes model. Am. J. Transpl. 20, 1551–1561. 10.1111/ajt.15812 32031745

[B90] Lebreton F. Hanna R. Wassmer C. H. Bellofatto K. Perez L. Othenin-Girard V. (2022). Mechanisms of immunomodulation and cytoprotection conferred to pancreatic islet by human amniotic epithelial cells. Stem Cell Rev. Rep. 18, 346–359. 10.1007/s12015-021-10269-w 34613550 PMC8799589

[B91] Lee B. E. Lee B. J. Lee K. J. Lee M. Lim Y. J. Choi J. K. (2022). A simple and efficient cryopreservation method for mouse small intestinal and colon organoids for regenerative medicine. Biochem. Biophys. Res. Commun. 595, 14–21. 10.1016/j.bbrc.2021.12.021 35093635

[B92] Lee J. H. Kim S. K. Khawar I. A. Jeong S. Y. Chung S. Kuh H. J. (2018a). Microfluidic co-culture of pancreatic tumor spheroids with stellate cells as a novel 3D model for investigation of stroma-mediated cell motility and drug resistance. J. Exp. Clin. Cancer Res. 37, 4. 10.1186/s13046-017-0654-6 29329547 PMC5767067

[B93] Lee K. K. Mccauley H. A. Broda T. R. Kofron M. J. Wells J. M. Hong C. I. (2018b). Human stomach-on-a-chip with luminal flow and peristaltic-like motility. Lab. Chip 18, 3079–3085. 10.1039/c8lc00910d 30238091 PMC6364752

[B94] Lee S. Y. Teng Y. Son M. Ku B. Hwang H. J. Tergaonkar V. (2021). Three-dimensional aggregated spheroid model of hepatocellular carcinoma using a 96-pillar/well plate. Molecules 26, 4949. 10.3390/molecules26164949 34443536 PMC8399878

[B95] Lee H. Chae S. Kim J. Y. Han W. Kim J. Choi Y. (2019). Cell-printed 3D liver-on-a-chip possessing a liver microenvironment and biliary system. Biofabrication 11, 025001. 10.1088/1758-5090/aaf9fa 30566930

[B96] Li N. T. Wu N. C. Cao R. Cadavid J. L. Latour S. Lu X. (2022). An off-the-shelf multi-well scaffold-supported platform for tumour organoid-based tissues. Biomaterials 291, 121883. 10.1016/j.biomaterials.2022.121883 36343611

[B97] Licata J. P. Schwab K. H. Har-EL Y. E. Gerstenhaber J. A. Lelkes P. I. (2023). Bioreactor technologies for enhanced organoid culture. Int. J. Mol. Sci. 24, 11427. 10.3390/ijms241411427 37511186 PMC10380004

[B98] Li H. Guo L. Wang C. Hu X. Xu Y. (2023a). Improving the value of molecular testing: current status and opportunities in colorectal cancer precision medicine. Cancer Biol. Med. 21, 21–28. 10.20892/j.issn.2095-3941.2023.0293 38038341 PMC10875284

[B99] Li L. Knutsdottir H. Hui K. Weiss M. J. He J. Philosophe B. (2019). Human primary liver cancer organoids reveal intratumor and interpatient drug response heterogeneity. JCI Insight 4, e121490. 10.1172/jci.insight.121490 30674722 PMC6413833

[B100] Liu X. Cheng Y. Abraham J. M. Wang Z. Wang Z. Ke X. (2018). Modeling Wnt signaling by CRISPR-Cas9 genome editing recapitulates neoplasia in human Barrett epithelial organoids. Cancer Lett. 436, 109–118. 10.1016/j.canlet.2018.08.017 30144514 PMC6152930

[B101] Liu Y. Gu X. Xuan M. Lou N. Fu L. Li J. (2024). Notch signaling in digestive system cancers: roles and therapeutic prospects. Cell Signal 124, 111476. 10.1016/j.cellsig.2024.111476 39428027

[B102] Liu Y. Li N. Zhu Y. (2023). Pancreatic organoids: a frontier method for investigating pancreatic-related diseases. Int. J. Mol. Sci. 24, 4027. 10.3390/ijms24044027 36835437 PMC9959977

[B103] Li X. Zhu H. Gu B. Yao C. Gu Y. Xu W. (2023b). Advancing intelligent organ-on-a-chip systems with comprehensive *in situ* bioanalysis. Adv. Mater 36, e2305268. 10.1002/adma.202305268 37688520

[B104] Li Y. Yuan K. Deng C. Tang H. Wang J. Dai X. (2024). Biliary stents for active materials and surface modification: recent advances and future perspectives. Bioact. Mater 42, 587–612. 10.1016/j.bioactmat.2024.08.031 39314863 PMC11417150

[B105] Magré L. Verstegen M. M. A. Buschow S. VAN DER Laan L. J. W. Peppelenbosch M. Desai J. (2023). Emerging organoid-immune co-culture models for cancer research: from oncoimmunology to personalized immunotherapies. J. Immunother. Cancer 11, e006290. 10.1136/jitc-2022-006290 37220953 PMC10231025

[B106] Maier C. F. Zhu L. Nanduri L. K. KüHN D. Kochall S. Thepkaysone M. L. (2021). Patient-derived organoids of cholangiocarcinoma. Int. J. Mol. Sci. 22, 8675. 10.3390/ijms22168675 34445380 PMC8395494

[B107] Majid Q. A. Ghimire B. R. Merkely B. Randi A. M. Harding S. E. Talman V. (2024). Generation and characterisation of scalable and stable human pluripotent stem cell-derived microvascular-like endothelial cells for cardiac applications. Angiogenesis 27, 561–582. 10.1007/s10456-024-09929-5 38775849 PMC11303486

[B108] Mandal A. Chatterjee K. (2024). 4D printing for biomedical applications. J. Mater Chem. B 12, 2985–3005. 10.1039/d4tb00006d 38436200

[B109] Marques I. A. Fernandes C. Tavares N. T. Pires A. S. Abrantes A. M. Botelho M. F. (2022). Magnetic-based human tissue 3D cell culture: a systematic review. Int. J. Mol. Sci. 23, 12681. 10.3390/ijms232012681 36293537 PMC9603906

[B110] Matano M. Date S. Shimokawa M. Takano A. Fujii M. Ohta Y. (2015). Modeling colorectal cancer using CRISPR-Cas9-mediated engineering of human intestinal organoids. Nat. Med. 21, 256–262. 10.1038/nm.3802 25706875

[B111] Mattei C. Lim R. Drury H. Nasr B. Li Z. Tadros M. A. (2019). Generation of vestibular tissue-like organoids from human pluripotent stem cells using the rotary cell culture system. Front. Cell Dev. Biol. 7, 25. 10.3389/fcell.2019.00025 30891447 PMC6413170

[B112] Mccarron S. Bathon B. Conlon D. M. Abbey D. Rader D. J. Gawronski K. (2021). Functional characterization of organoids derived from irreversibly damaged liver of patients with NASH. Hepatology 74, 1825–1844. 10.1002/hep.31857 33901295 PMC12928191

[B113] Mccracken K. W. Catá E. M. Crawford C. M. Sinagoga K. L. Schumacher M. Rockich B. E. (2014). Modelling human development and disease in pluripotent stem-cell-derived gastric organoids. Nature 516, 400–404. 10.1038/nature13863 25363776 PMC4270898

[B114] Miao Y. Ha A. DE Lau W. Yuki K. Santos A. J. M. You C. (2020). Next-generation surrogate wnts support organoid growth and deconvolute frizzled pleiotropy *in vivo* . Cell Stem Cell 27, 840–851. 10.1016/j.stem.2020.07.020 32818433 PMC7655723

[B115] Molakandov K. Berti D. A. Beck A. Elhanani O. Walker M. D. Soen Y. (2021). Selection for CD26(-) and CD49A(+) cells from pluripotent stem cells-derived islet-like clusters improves therapeutic activity in diabetic mice. Front. Endocrinol. (Lausanne) 12, 635405. 10.3389/fendo.2021.635405 34025576 PMC8131825

[B116] Monteduro A. G. Rizzato S. Caragnano G. Trapani A. Giannelli G. Maruccio G. (2023). Organs-on-chips technologies - a guide from disease models to opportunities for drug development. Biosens. Bioelectron. 231, 115271. 10.1016/j.bios.2023.115271 37060819

[B117] Moreira L. Bakir B. Chatterji P. Dantes Z. Reichert M. Rustgi A. K. (2018). Pancreas 3D organoids: current and future aspects as a research platform for personalized medicine in pancreatic cancer. Cell. Mol. Gastroenterology Hepatology 5, 289–298. 10.1016/j.jcmgh.2017.12.004 PMC5849862 29541683

[B118] Nagle P. W. Hosper N. A. Ploeg E. M. VAN Goethem M. J. Brandenburg S. Langendijk J. A. (2016). The *in vitro* response of tissue stem cells to irradiation with different linear energy transfers. Int. J. Radiat. Oncol. Biol. Phys. 95, 103–111. 10.1016/j.ijrobp.2016.02.020 27084633

[B119] Nakagawa H. Kasagi Y. Karakasheva T. A. Hara T. Aaron B. Shimonosono M. (2020). Modeling epithelial homeostasis and reactive epithelial changes in human and murine three-dimensional esophageal organoids. Curr. Protoc. Stem Cell Biol. 52, e106. 10.1002/cpsc.106 32105412 PMC7288850

[B120] Nanki K. Toshimitsu K. Takano A. Fujii M. Shimokawa M. Ohta Y. (2018). Divergent routes toward wnt and R-spondin niche independency during human gastric carcinogenesis. Cell 174, 856–869. 10.1016/j.cell.2018.07.027 30096312

[B121] Nasiri R. Zhu Y. DE Barros N. R. (2024). Microfluidics and organ-on-a-chip for disease modeling and drug screening. Biosens. (Basel) 14, 86. 10.3390/bios14020086 PMC10887020 38392005

[B122] Nevola R. Beccia D. Rosato V. Ruocco R. Mastrocinque D. Villani A. (2023). HBV infection and host interactions: the role in viral persistence and oncogenesis. Int. J. Mol. Sci. 24, 7651. 10.3390/ijms24087651 37108816 PMC10145402

[B123] Nikokiraki C. Psaraki A. Roubelakis M. G. (2022). The potential clinical use of stem/progenitor cells and organoids in liver diseases. Cells 11, 1410. 10.3390/cells11091410 35563716 PMC9101582

[B124] Nowicki-Osuch K. Zhuang L. Jammula S. Bleaney C. W. Mahbubani K. T. Fitzgerald R. C. (2021). Molecular phenotyping reveals the identity of Barrett's esophagus and its malignant transition. Science 373, 760–767. 10.1126/science.abd1449 34385390

[B125] O'Connell L. Winter D. C. (2020). Organoids: past learning and future directions. Stem Cells Dev. 29, 281–289. 10.1089/scd.2019.0227 31805828

[B126] Ooft S. N. Weeber F. Dijkstra K. K. Mclean C. M. Kaing S. VAN Werkhoven E. (2019). Patient-derived organoids can predict response to chemotherapy in metastatic colorectal cancer patients. Sci. Transl. Med. 11, eaay2574. 10.1126/scitranslmed.aay2574 31597751

[B127] Ouchi R. Togo S. Kimura M. Shinozawa T. Koido M. Koike H. (2019). Modeling steatohepatitis in humans with pluripotent stem cell-derived organoids. Cell Metab. 30, 374–384. 10.1016/j.cmet.2019.05.007 31155493 PMC6687537

[B128] Ouyang S. Li H. Lou L. Huang Q. Zhang Z. Mo J. (2022). Inhibition of STAT3-ferroptosis negative regulatory axis suppresses tumor growth and alleviates chemoresistance in gastric cancer. Redox Biol. 52, 102317. 10.1016/j.redox.2022.102317 35483272 PMC9108091

[B129] Özkan A. Logrande N. T. Feitor J. F. Goyal G. Ingber D. E. (2024). Intestinal organ chips for disease modelling and personalized medicine. Nat. Rev. Gastroenterology and Hepatology 21, 751–773. 10.1038/s41575-024-00968-3 39192055

[B130] Palasantzas V. Tamargo-Rubio I. LE K. Slager J. Wijmenga C. Jonkers I. H. (2023). iPSC-derived organ-on-a-chip models for personalized human genetics and pharmacogenomics studies. Trends Genet. 39, 268–284. 10.1016/j.tig.2023.01.002 36746737

[B131] Parente I. A. Chiara L. Bertoni S. (2024). Exploring the potential of human intestinal organoids: applications, challenges, and future directions. Life Sci. 352, 122875. 10.1016/j.lfs.2024.122875 38942359

[B132] Pei Q. Yi Q. Tang L. (2023). Liver fibrosis resolution: from molecular mechanisms to therapeutic opportunities. Int. J. Mol. Sci. 24, 9671. 10.3390/ijms24119671 37298621 PMC10253436

[B133] Peters Y. AL-Kaabi A. Shaheen N. J. Chak A. Blum A. Souza R. F. (2019). Barrett oesophagus. Nat. Rev. Dis. Prim. 5, 35. 10.1038/s41572-019-0086-z 31123267

[B134] Pinho D. Santos D. Vila A. Carvalho S. (2021). Establishment of colorectal cancer organoids in microfluidic-based system. Micromachines (Basel) 12, 497. 10.3390/mi12050497 33924829 PMC8146416

[B135] Piraino F. Costa M. Meyer M. Cornish G. Ceroni C. Garnier V. (2024). Organoid models: the future companions of personalized drug development. Biofabrication 16, 032009. 10.1088/1758-5090/ad3e30 38608454

[B136] Poling H. M. Sundaram N. Fisher G. W. Singh A. Shiley J. R. Nattamai K. (2024). Human pluripotent stem cell-derived organoids repair damaged bowel *in vivo* . Cell Stem Cell 31, 1513–1523.e7. 10.1016/j.stem.2024.08.009 39270642 PMC11824906

[B137] Pradhan S. Karve S. S. Weiss A. A. Hawkins J. Poling H. M. Helmrath M. A. (2020). Tissue responses to Shiga toxin in human intestinal organoids. Cell Mol. Gastroenterol. Hepatol. 10, 171–190. 10.1016/j.jcmgh.2020.02.006 32145469 PMC7240222

[B138] Price S. Bhosle S. GonçALVES E. Li X. Mcclurg D. P. Barthorpe S. (2022). A suspension technique for efficient large-scale cancer organoid culturing and perturbation screens. Sci. Rep. 12, 5571. 10.1038/s41598-022-09508-y 35368031 PMC8976852

[B139] Ren X. Huang M. Weng W. Xie Y. Wu Y. Zhu S. (2023). Personalized drug screening in patient-derived organoids of biliary tract cancer and its clinical application. Cell Rep. Med. 4, 101277. 10.1016/j.xcrm.2023.101277 37944531 PMC10694672

[B140] Reza H. A. Okabe R. Takebe T. (2021). Organoid transplant approaches for the liver. Transpl. Int. 34, 2031–2045. 10.1111/tri.14128 34614263 PMC8602742

[B141] Ringel T. Frey N. Ringnalda F. Janjuha S. Cherkaoui S. Butz S. (2020). Genome-scale CRISPR screening in human intestinal organoids identifies drivers of TGF-β resistance. Cell Stem Cell 26, 431–440. 10.1016/j.stem.2020.02.007 32142663

[B142] Rosowski J. BräUNIG J. Amler A. K. Strietzel F. P. Lauster R. Rosowski M. (2019). Emulating the early phases of human tooth development *in vitro* . Sci. Rep. 9, 7057. 10.1038/s41598-019-43468-0 31065008 PMC6505527

[B143] Ryu N. E. Lee S. H. Park H. (2019). Spheroid culture system methods and applications for mesenchymal stem cells. Cells 8, 1620. 10.3390/cells8121620 31842346 PMC6953111

[B144] Sada R. Yamamoto H. Matsumoto S. Harada A. Kikuchi A. (2024). Newly developed humanized anti-CKAP4 antibody suppresses pancreatic cancer growth by inhibiting DKK1-CKAP4 signaling. Cancer Sci. 115, 3358–3369. 10.1111/cas.16278 39118263 PMC11447883

[B145] Sampaziotis F. Muraro D. Tysoe O. C. Sawiak S. Beach T. E. Godfrey E. M. (2021). Cholangiocyte organoids can repair bile ducts after transplantation in the human liver. Science 371, 839–846. 10.1126/science.aaz6964 33602855 PMC7610478

[B146] Saorin G. Caligiuri I. Rizzolio F. (2023). Microfluidic organoids-on-a-chip: the future of human models. Semin. Cell Dev. Biol. 144, 41–54. 10.1016/j.semcdb.2022.10.001 36241560

[B147] Sato T. Stange D. E. Ferrante M. Vries R. G. VAN Es J. H. VAN Den Brink S. (2011). Long-term expansion of epithelial organoids from human colon, adenoma, adenocarcinoma, and Barrett's epithelium. Gastroenterology 141, 1762–1772. 10.1053/j.gastro.2011.07.050 21889923

[B148] Schnalzger T. E. DE Groot M. H. Zhang C. Mosa M. H. Michels B. E. RöDER J. (2019). 3D model for CAR-mediated cytotoxicity using patient-derived colorectal cancer organoids. Embo J. 38, e100928. 10.15252/embj.2018100928 31036555 PMC6576164

[B149] Schumacher M. A. Aihara E. Feng R. Engevik A. Shroyer N. F. Ottemann K. M. (2015). The use of murine-derived fundic organoids in studies of gastric physiology. J. Physiol. 593, 1809–1827. 10.1113/jphysiol.2014.283028 25605613 PMC4405744

[B150] Serra D. Mayr U. Boni A. Lukonin I. Rempfler M. Challet Meylan L. (2019). Self-organization and symmetry breaking in intestinal organoid development. Nature 569, 66–72. 10.1038/s41586-019-1146-y 31019299 PMC6544541

[B151] Serrano Martinez P. Cinat D. VAN Luijk P. Baanstra M. DE Haan G. Pringle S. (2021). Mouse parotid salivary gland organoids for the *in vitro* study of stem cell radiation response. Oral Dis. 27, 52–63. 10.1111/odi.13475 32531849 PMC7818507

[B152] Shoji J. Y. Davis R. P. Mummery C. L. Krauss S. (2023). Global literature analysis of organoid and organ-on-chip research. Adv. Healthc. Mater 13, e2301067. 10.1002/adhm.202301067 37479227

[B153] Singh A. Poling H. M. Spence J. R. Wells J. M. Helmrath M. A. (2020). Gastrointestinal organoids: a next-generation tool for modeling human development. Am. J. Physiol. Gastrointest. Liver Physiol. 319, G375–G381. 10.1152/ajpgi.00199.2020 32658619 PMC7509262

[B154] Sontheimer-Phelps A. Hassell B. A. Ingber D. E. (2019). Modelling cancer in microfluidic human organs-on-chips. Nat. Rev. Cancer 19, 65–81. 10.1038/s41568-018-0104-6 30647431

[B155] Spurrier R. G. Speer A. L. Hou X. EL-Nachef W. N. Grikscheit T. C. (2015). Murine and human tissue-engineered esophagus form from sufficient stem/progenitor cells and do not require microdesigned biomaterials. Tissue Eng. Part A 21, 906–915. 10.1089/ten.TEA.2014.0357 25298083 PMC4356247

[B156] Su H. Li Q. Li D. Li H. Feng Q. Cao X. (2022). A versatile strategy to construct free-standing multi-furcated vessels and a complicated vascular network in heterogeneous porous scaffolds via combination of 3D printing and stimuli-responsive hydrogels. Mater Horiz. 9, 2393–2407. 10.1039/d2mh00314g 35789239

[B157] Sun B. Zhao Y. Wu W. Zhao Q. Li G. (2021). A superhydrophobic chip integrated with an array of medium reservoirs for long-term hanging drop spheroid culture. Acta Biomater. 135, 234–242. 10.1016/j.actbio.2021.08.006 34389482

[B158] Tabatabaei Rezaei N. Kumar H. Liu H. Rajeev A. Natale G. Lee S. (2024). 3D bioprinting of liver microenvironment model using photocrosslinkable decellularized extracellular matrix based hydrogel.

[B159] Tabe S. Takeuchi K. Aoshima K. Okumura A. Yamamoto Y. Yanagisawa K. (2025). A pancreatic cancer organoid incorporating macrophages reveals the correlation between the diversity of tumor-associated macrophages and cancer cell survival. Biomaterials 314, 122838. 10.1016/j.biomaterials.2024.122838 39348736

[B160] Tanaka N. Osman A. A. Takahashi Y. Lindemann A. Patel A. A. Zhao M. (2018). Head and neck cancer organoids established by modification of the CTOS method can be used to predict *in vivo* drug sensitivity. Oral Oncol. 87, 49–57. 10.1016/j.oraloncology.2018.10.018 30527243 PMC6294331

[B161] Tang X. Y. Wu S. Wang D. Chu C. Hong Y. Tao M. (2022). Human organoids in basic research and clinical applications. Signal Transduct. Target Ther. 7, 168. 10.1038/s41392-022-01024-9 35610212 PMC9127490

[B162] Tepe U. Aslanbay Guler B. Imamoglu E. (2023). Applications and sensory utilizations of magnetic levitation in 3D cell culture for tissue Engineering. Mol. Biol. Rep. 50, 7017–7025. 10.1007/s11033-023-08585-0 37378748

[B163] Testa G. Mainardi M. Vannini E. Pancrazi L. Cattaneo A. Costa M. (2022). Disentangling the signaling complexity of nerve growth factor receptors by CRISPR/Cas9. FASEB J. 36, e22498. 10.1096/fj.202101760RR 37036720

[B164] Thrift A. P. (2021). Global burden and epidemiology of Barrett oesophagus and oesophageal cancer. Nat. Rev. Gastroenterol. Hepatol. 18, 432–443. 10.1038/s41575-021-00419-3 33603224

[B165] Tigani E. K. Skrtic D. Valerio M. S. Kaufman G. (2019). Assessing the effect of triethyleneglycol dimethacrylate on tissue repair in 3D organotypic cultures. J. Appl. Toxicol. 39, 247–259. 10.1002/jat.3714 30229966

[B166] Tiriac H. Belleau P. Engle D. D. Plenker D. DeschêNES A. Somerville T. D. D. (2018). Organoid profiling identifies common responders to chemotherapy in pancreatic cancer. Cancer Discov. 8, 1112–1129. 10.1158/2159-8290.CD-18-0349 29853643 PMC6125219

[B167] Tosca E. M. Ronchi D. Facciolo D. Magni P. (2023). Replacement, reduction, and refinement of animal experiments in anticancer drug development: the contribution of 3D *in vitro* cancer models in the drug efficacy assessment. Biomedicines 11, 1058. 10.3390/biomedicines11041058 37189676 PMC10136119

[B168] Tsang K. Y. Fantini M. Mavroukakis S. A. Zaki A. Annunziata C. M. Arlen P. M. (2022). Development and characterization of an anti-cancer monoclonal antibody for treatment of human carcinomas. Cancers (Basel) 14, 3037. 10.3390/cancers14133037 35804808 PMC9264992

[B169] Ueno T. Wada M. Hoshino K. Uemoto S. Taguchi T. Furukawa H. (2013). Impact of pediatric intestinal transplantation on intestinal failure in Japan: findings based on the Japanese intestinal transplant registry. Pediatr. Surg. Int. 29, 1065–1070. 10.1007/s00383-013-3392-7 23982390

[B170] VAN DE Wetering M. Francies H. E. Francis J. M. Bounova G. Iorio F. Pronk A. (2015). Prospective derivation of a living organoid biobank of colorectal cancer patients. Cell 161, 933–945. 10.1016/j.cell.2015.03.053 25957691 PMC6428276

[B171] Verissimo C. S. Overmeer R. M. Ponsioen B. Drost J. Mertens S. Verlaan-Klink I. (2016). Targeting mutant RAS in patient-derived colorectal cancer organoids by combinatorial drug screening. Elife 5, e18489. 10.7554/eLife.18489 27845624 PMC5127645

[B172] Verstegen M. M. A. Roos F. J. M. Burka K. Gehart H. Jager M. DE Wolf M. (2020). Human extrahepatic and intrahepatic cholangiocyte organoids show region-specific differentiation potential and model cystic fibrosis-related bile duct disease. Sci. Rep. 10, 21900. 10.1038/s41598-020-79082-8 33318612 PMC7736890

[B173] Vijftigschild L. A. Berkers G. Dekkers J. F. Zomer-VAN Ommen D. D. Matthes E. Kruisselbrink E. (2016). β2-Adrenergic receptor agonists activate CFTR in intestinal organoids and subjects with cystic fibrosis. Eur. Respir. J. 48, 768–779. 10.1183/13993003.01661-2015 27471203

[B174] Vlachogiannis G. Hedayat S. Vatsiou A. Jamin Y. FernáNDEZ-Mateos J. Khan K. (2018). Patient-derived organoids model treatment response of metastatic gastrointestinal cancers. Science 359, 920–926. 10.1126/science.aao2774 29472484 PMC6112415

[B175] Wang Q. Guo F. Jin Y. Ma Y. (2022). Applications of human organoids in the personalized treatment for digestive diseases. Signal Transduct. Target Ther. 7, 336. 10.1038/s41392-022-01194-6 36167824 PMC9513303

[B176] Wang S. Wang Y. Xun X. Zhang C. Xiang X. Cheng Q. (2020). Hedgehog signaling promotes sorafenib resistance in hepatocellular carcinoma patient-derived organoids. J. Exp. Clin. Cancer Res. 39, 22. 10.1186/s13046-020-1523-2 31992334 PMC6986013

[B177] Wang X. Serrano Martinez P. Terpstra J. H. Shaalan A. Proctor G. B. Spijkervet F. K. L. (2021). β-Adrenergic signaling induces Notch-mediated salivary gland progenitor cell control. Stem Cell Rep. 16, 2813–2824. 10.1016/j.stemcr.2021.09.015 PMC8581054 34678204

[B178] Wan X. Xiao Z. Tian Y. Chen M. Liu F. Wang D. (2024). Recent advances in 4D printing of advanced materials and structures for functional applications. Adv. Mater 36, e2312263. 10.1002/adma.202312263 38439193

[B179] Watanabe S. Yogo A. Otsubo T. Umehara H. Oishi J. Kodo T. (2022). Establishment of patient-derived organoids and a characterization-based drug discovery platform for treatment of pancreatic cancer. BMC Cancer 22, 489. 10.1186/s12885-022-09619-9 35505283 PMC9063137

[B180] Wu J. Chen C. Qin C. Li Y. Jiang N. Yuan Q. (2023a). Mimicking the biological sense of taste *in vitro* using a taste organoids-on-a-chip system. Adv. Sci. (Weinh) 10, e2206101. 10.1002/advs.202206101 36638268 PMC9982573

[B181] Wu Y. Ye W. Gao Y. Yi Z. Chen Z. Qu C. (2023b). Application of organoids in regenerative medicine. Stem Cells 41, 1101–1112. 10.1093/stmcls/sxad072 37724396

[B182] Xing C. Kemas A. Mickols E. Klein K. Artursson P. Lauschke V. M. (2024). The choice of ultra-low attachment plates impacts primary human and primary canine hepatocyte spheroid formation, phenotypes, and function. Biotechnol. J. 19, e2300587. 10.1002/biot.202300587 38403411

[B183] Xu H. Lyu X. Yi M. Zhao W. Song Y. Wu K. (2018). Organoid technology and applications in cancer research. J. Hematol. Oncol. 11, 116. 10.1186/s13045-018-0662-9 30219074 PMC6139148

[B184] Yan H. H. N. Siu H. C. Law S. Ho S. L. Yue S. S. K. Tsui W. Y. (2018). A comprehensive human gastric cancer organoid biobank captures tumor subtype heterogeneity and enables therapeutic screening. Cell Stem Cell 23, 882–897. 10.1016/j.stem.2018.09.016 30344100

[B185] Yang L. Chen J. Liang J. Zhang Y. Wang Q. Ren X. (2022). Modeling hepatoblastoma development with human fetal liver organoids reveals YAP1 activation is sufficient for tumorigenesis. Protein Cell 13, 683–688. 10.1007/s13238-021-00893-0 34893955 PMC9233724

[B186] Yang R. Yu Y. (2023). Patient-derived organoids in translational oncology and drug screening. Cancer Lett. 562, 216180. 10.1016/j.canlet.2023.216180 37061121

[B187] Yao Y. Xu X. Yang L. Zhu J. Wan J. Shen L. (2020). Patient-derived organoids predict chemoradiation responses of locally advanced rectal cancer. Cell Stem Cell 26, 17–26. 10.1016/j.stem.2019.10.010 31761724

[B188] Ye S. Marsee A. VAN Tienderen G. S. Rezaeimoghaddam M. Sheikh H. Samsom R. A. (2024). Accelerated production of human epithelial organoids in a miniaturized spinning bioreactor. Cell Rep. Methods 4, 100903. 10.1016/j.crmeth.2024.100903 39561715 PMC11705766

[B189] Yin Y. B. DE Jonge H. R. Wu X. Yin Y. L. (2019). Mini-gut: a promising model for drug development. Drug Discov. Today 24, 1784–1794. 10.1016/j.drudis.2019.06.006 31212027

[B190] Yin J. Meng H. Lin J. Ji W. Xu T. Liu H. (2022). Pancreatic islet organoids-on-a-chip: how far have we gone? J. Nanobiotechnology 20, 308. 10.1186/s12951-022-01518-2 35764957 PMC9238112

[B191] Yin Y. Chen S. Hakim M. S. Wang W. Xu L. Dang W. (2018). 6-Thioguanine inhibits rotavirus replication through suppression of Rac1 GDP/GTP cycling. Antivir. Res. 156, 92–101. 10.1016/j.antiviral.2018.06.011 29920300 PMC7113846

[B192] Yoshihara E. O'Connor C. Gasser E. Wei Z. Oh T. G. Tseng T. W. (2020). Immune-evasive human islet-like organoids ameliorate diabetes. Nature 586, 606–611. 10.1038/s41586-020-2631-z 32814902 PMC7872080

[B193] Yu Y. Y. Zhu Y. J. Xiao Z. Z. Chen Y. D. Chang X. S. Liu Y. H. (2022). The pivotal application of patient-derived organoid biobanks for personalized treatment of gastrointestinal cancers. Biomark. Res. 10, 73. 10.1186/s40364-022-00421-0 36207749 PMC9547471

[B194] Yuan B. Zhao X. Wang X. Liu E. Liu C. Zong Y. (2022). Patient-derived organoids for personalized gallbladder cancer modelling and drug screening. Clin. Transl. Med. 12, e678. 10.1002/ctm2.678 35075805 PMC8786696

[B195] Zeiringer S. Wiltschko L. Glader C. Reiser M. Absenger-Novak M. FröHLICH E. (2023). Development and characterization of an *in vitro* intestinal model including extracellular matrix and macrovascular endothelium. Mol. Pharm. 20, 5173–5184. 10.1021/acs.molpharmaceut.3c00532 37677739 PMC10548470

[B196] Zhang S. Sui Y. Yan S. Zhang Y. Ding C. Su X. (2022). Retinoic acid and FGF10 promote the differentiation of pluripotent stem cells into salivary gland placodes. Stem Cell Res. Ther. 13, 368. 10.1186/s13287-022-03033-5 35902913 PMC9330698

[B197] Zhang Y. Que J. (2020). BMP signaling in development, stem cells, and diseases of the gastrointestinal tract. Annu. Rev. Physiol. 82, 251–273. 10.1146/annurev-physiol-021119-034500 31618602 PMC13090088

[B198] Zhang Y. Yang Y. Jiang M. Huang S. X. Zhang W. AL Alam D. (2018). 3D modeling of esophageal development using human PSC-derived basal progenitors reveals a critical role for Notch signaling. Cell Stem Cell 23, 516–529. 10.1016/j.stem.2018.08.009 30244870 PMC6282026

[B199] Zhang Y. Zhang C. Peng C. Jia J. (2024). Unraveling the crosstalk: circRNAs and the wnt signaling pathway in cancers of the digestive system. Noncoding RNA Res. 9, 853–864. 10.1016/j.ncrna.2024.03.004 38586314 PMC10995981

[B200] Zhao B. Chen Y. Jiang N. Yang L. Sun S. Zhang Y. (2019a). Znhit1 controls intestinal stem cell maintenance by regulating H2A.Z incorporation. Nat. Commun. 10, 1071. 10.1038/s41467-019-09060-w 30842416 PMC6403214

[B201] Zhao H. Hu C. Y. Chen W. M. Huang P. (2019b). Lactate promotes cancer stem-like property of oral sequamous cell carcinoma. Curr. Med. Sci. 39, 403–409. 10.1007/s11596-019-2050-2 31209810

[B202] Zhao H. Jiang E. Shang Z. (2021). 3D Co-culture of cancer-associated fibroblast with oral cancer organoids. J. Dent. Res. 100, 201–208. 10.1177/0022034520956614 32881601

[B203] Zhao H. Li R. Chen Y. Yang X. Shang Z. (2023). Stromal nicotinamide N-methyltransferase orchestrates the crosstalk between fibroblasts and tumour cells in oral squamous cell carcinoma: evidence from patient-derived assembled organoids. Oncogene 42, 1166–1180. 10.1038/s41388-023-02642-5 36823377

[B204] Zhou L. Luo D. Lu W. Han J. Zhao M. Li X. (2024a). Gastrointestinal tract organoids as novel tools in drug discovery. Front. Pharmacol. 15, 1463114. 10.3389/fphar.2024.1463114 39281285 PMC11394194

[B205] Zhou S. Yang J. Li R. Chen Y. Li C. Chen C. (2023). Live imaging of 3D hanging drop arrays through manipulation of light-responsive pyroelectric slippery surface and chip adhesion. Nano Lett. 23, 10710–10718. 10.1021/acs.nanolett.3c02570 38010943

[B206] Zhou T. Hou X. Yan J. Li L. Xie Y. Bai W. (2024b). CD64(+) fibroblast-targeted vilanterol and a STING agonist augment CLDN18.2 BiTEs efficacy against pancreatic cancer by reducing desmoplasia and enriching stem-like CD8(+) T cells. Gut 73, 1984–1998. 10.1136/gutjnl-2024-332371 39187291

[B207] Zou J. Wang S. Chai N. Yue H. Ye P. Guo P. (2022). Construction of gastric cancer patient-derived organoids and their utilization in a comparative study of clinically used paclitaxel nanoformulations. J. Nanobiotechnology 20, 233. 10.1186/s12951-022-01431-8 35585597 PMC9118843

